# AI-Enabled First-Response Support After Sexual and Gender-Based Violence: A PRISMA-ScR Scoping Review

**DOI:** 10.3390/healthcare14142174

**Published:** 2026-07-18

**Authors:** Paolo Bailo, Chiara Carsana, Maria Garreffa, Anna Carannante, Marco Giustini, Cecilia Fazio, Loredana Falzano, Andrea Piccinini, Simona Gaudi

**Affiliations:** 1 Section of Legal Medicine, School of Law, University of Camerino, 62032 Camerino, Italy; paolo.bailo@unicam.it; 2 Section of Legal Medicine and Insurance Medicine, Department of Biomedical Sciences for Health, University of Milan, 20122 Milan, Italy; chiara.carsana1@unimi.it (C.C.); maria.garreffa@unimi.it (M.G.); 3 Department of Environment and Health, Italian Institute of Health, 00161 Rome, Italy; anna.carannante@iss.it (A.C.); marco.giustini@iss.it (M.G.); cecilia.fazio@iss.it (C.F.); simona.gaudi@iss.it (S.G.); 4 National Centre for Global Health, Italian Institute of Health, 00161 Rome, Italy

**Keywords:** sexual violence, gender-based violence, domestic violence, intimate partner violence, artificial intelligence, crisis hotlines, chatbots, triage, scoping review

## Abstract

**Highlights:**

**What are the main findings?**

- AI-enabled first-response tools after sexual and gender-based violence are emerging mainly as entry-layer supports for chatbots, clinical flagging, emergency-department narrative surveillance, online disclosure triage, legal/support routing, and digital reporting or safety-navigation tools.
- The evidence remains upstream of service impact: most sources report technical performance, feasibility, usability, acceptability, or systems-audit findings, while escalation quality, safety monitoring, referral uptake, and survivor-centred outcomes remain rarely measured.

**What are the implications of the main findings?**

- Future studies should evaluate AI tools as components of violence-response pathways, not only as models or interfaces, with explicit measurement of human handover, service routing, adverse events, and downstream support outcomes.
- Safe implementation requires trauma-informed boundaries, trace-safety under coercive control, equity and portability testing, accountable human oversight, and clear procedures for escalation, failure or unsafe outputs.

**Abstract:**

Background: Artificial intelligence (AI) is increasingly proposed to augment early-stage assistance for survivors of sexual and gender-based violence (GBV), including intimate partner and domestic violence, across crisis hotlines, specialist services, digital reporting channels, legal support tools and healthcare pathways. However, the scope, maturity and evaluative strength of the peer-reviewed evidence remain uncertain. We aimed to map the application domains, evaluative maturity, and implementation and safety gaps of this evidence base. Methods: We conducted a scoping review reported according to the PRISMA Extension for Scoping Reviews (PRISMA-ScR), using a Population–Concept–Context framework focused on AI-enabled first-response and early support. Searches in Scopus, Web of Science Core Collection and PubMed were supplemented by targeted searches of IEEE Xplore and ACM Digital Library. Records were screened against predefined criteria, charted using a structured form and synthesised descriptively. Results: Original searches yielded 187 records and 21 included sources of evidence. The supplementary search identified 539 records/candidates; 27 full texts were assessed and 6 additional sources met eligibility criteria, yielding 27 included sources of evidence. Evidence covered survivor-facing conversational support; screening and triage in emergency and specialist services; social-triage and online disclosure models; survivor-informed help-seeking and chatbot design; legal/support routing; and enabling modalities such as speech-based approaches. Most sources reported technical performance, usability, acceptability or systems-audit findings, while no workflow-integrated evaluation was identified and survivor-centred effectiveness outcomes, service uptake and adverse-event monitoring were rarely reported. Conclusions: The evidence remains heterogeneous and early-stage, with limited support for service-integrated effectiveness or safety. Included sources more often assessed models, interfaces or prototypes than downstream pathway outcomes. The findings support cautious, pathway-aware interpretation and identify recurring concerns regarding escalation, accountability, equity, digital trace safety and human handover. The proposed practice considerations and outcome domains are author-informed priorities for future pilot and implementation studies, not validated guidelines.

## 1. Introduction

Sexual violence and gender-based violence (GBV), including intimate partner violence (IPV) and domestic violence, create a distinctive first-contact problem for health and victim support services. Survivors often require immediate, trauma-informed guidance precisely when disclosure is most challenging, safety is uncertain, and pathways across crisis, healthcare, legal and specialist services are fragmented [1,2]. Crisis hotlines, anti-violence services, emergency departments (EDs), specialist referral centres and legal support pathways therefore act as critical gateways, yet they are frequently constrained by limited capacity, uneven availability of trained personnel, and discontinuities between initial disclosure, rapid risk appraisal, and longer-term support [3,4]. In practice, the earliest steps—making contact, articulating needs, being believed, being routed to the right service, and receiving actionable safety information—can determine whether a survivor obtains timely care and protection or disengages from support [5,6,7,8,9].

Artificial intelligence (AI) is increasingly presented as a candidate infrastructure for strengthening this first-contact layer [10,11,12,13,14,15,16,17]. In this review, the relevant innovation claim is not automating care or replacing advocates, clinicians or legal professionals. Rather, it concerns whether AI can safely augment the entry layer of support by helping services deliver consistent, scalable functions that are otherwise difficult to maintain across time and demand. Emerging applications target operational tasks such as identifying urgent risk signals in text or speech, classifying support needs, routing survivors towards appropriate resources, supporting structured intake or reporting, and providing bounded information while maintaining clear expectations and reliable escalation to human responders. This conceptualisation holds only if boundaries, handover and accountability are explicit and testable [12,18,19,20,21].

At the same time, violence-response contexts are not neutral deployment environments for AI. They are safety-critical and privacy-sensitive environments, frequently shaped by coercive control, surveillance, shared devices, and constrained digital autonomy [19]. These features raise the bar for safety-by-design beyond what is typically assumed in general health-chatbot deployments. A tool can be unsafe even when model-level performance appears strong if it fails to escalate under time pressure, generates content that encourages risky disclosure, leaves an exploitable digital trace, or degrades across populations and languages different from those represented in development datasets. The decision to map rather than pool evidence is consistent with scoping-review guidance and staged-evaluation frameworks for early innovation and AI systems [22,23,24,25]. These concerns are reinforced by broader digital-health and AI-governance frameworks, including evidence-tier approaches for digital health technologies, the European Union Artificial Intelligence Act, and World Health Organisation guidance on digital interventions as complements rather than substitutes for functioning health systems [26,27,28].

A scoping review is appropriate because the field is heterogeneous, early-stage, and distributed across the biomedical, engineering, human–computer interaction, legal and social service literature. The available studies differ substantially in target population, AI modality, data source, intended point in the pathway, and evaluation design. These features make meta-analysis or a conventional effectiveness-focused systematic review premature. The present review, therefore, maps what has been built or proposed, where AI components are intended to sit within survivor pathways, and which evaluation domains have and have not been measured.

This scoping review addresses that gap by mapping peer-reviewed evidence on AI-enabled tools designed to support early-stage assistance for survivors of sexual violence and GBV across first-response pathways. We characterise applications by service setting, AI modality, intended function, first-response relevance, and evaluation depth. We then synthesise how systems have been evaluated across technical performance, usability/acceptability, and implementation-relevant dimensions, and identify research and translation gaps that must be addressed before such tools can be interpreted as safe, equitable, service-integrated components of violence-response pathways.

To support translation beyond descriptive mapping, we also outline author-informed practice considerations for survivor-facing AI in first-response settings. These include an evidence maturity heuristic, minimum safety-oriented escalation considerations, an illustrative coercive-control threat model, and candidate outcome domains for future pilot and implementation studies. These elements are presented as practice-oriented proposals informed by the review and adjacent governance literature, not as consensus-derived guidelines or validated framework outputs.

## 2. Methods

### 2.1. Review Design and Reporting

We conducted a scoping review to map peer-reviewed evidence on AI-enabled tools intended to support early-stage assistance for survivors of sexual violence and GBV, including intimate partner violence and domestic violence, across crisis, first-response, legal support, and digitally mediated help-seeking pathways. The review was planned and reported with reference to the PRISMA Extension for Scoping Reviews (PRISMA-ScR) [22]. Methodological choices were informed by the foundational scoping-study framework described by Arksey and O’Malley [23]. No review protocol was prospectively registered, and no retrospective registration was undertaken or is planned. To strengthen transparency, the full search strategy, complete PRISMA-ScR checklist, data charting form, conceptual crosswalk for the M0–M3 heuristic, detailed extraction matrices, source-level coding matrix, supplementary full-text screening decisions, and final record accounting are provided in the Supplementary Materials.

### 2.2. Information Sources and Search Strategy

Original searches were completed on 16 June 2026 in Scopus, Web of Science Core Collection, and PubMed. The search strategy combined controlled vocabulary and free-text terms spanning three core components: sexual violence/GBV; first-response and victim service contexts; and AI methods and systems, including machine learning (ML), deep learning, natural language processing (NLP), large language models (LLMs), chatbots, speech recognition, and sentiment analysis. For each database, broad and narrow strategy variants were combined into a single master record set after export and deduplication. Complete database-specific strings are reported in the Supplementary Materials.

To improve coverage of the computer science and engineering literature, a targeted supplementary search was conducted on 9 July 2026 in IEEE Xplore and ACM Digital Library. IEEE Xplore records were exported in a structured format and deduplicated within the source. ACM Digital Library results were screened in-platform because bulk structured export was unavailable. Potentially eligible records were retained for full-text assessment, and the corresponding full-text decisions are reported in the Supplementary Materials. Residual overlap between the supplementary sources and the original search was identified during screening. The search did not apply language filters at retrieval. At the eligibility/full-text stage, inclusion was limited to English-language full texts because this was the language accessible for complete assessment by the review team. Grey literature, including operational reports, programmatic guidelines and hotline documentation, as well as regional databases, was not searched; the implications of this decision are addressed in the Limitations Section.

### 2.3. Eligibility Criteria

Eligibility criteria were defined in advance using a Population–Concept–Context framework. The population comprised survivors or victims of sexual violence and/or GBV. The concept of interest was an explicit AI component, broadly including ML, NLP, LLMs, conversational agents or chatbots, described as part of a tool intended to support survivor-facing assistance, triage, screening, reporting, prioritisation, legal/support routing, or clinician/service-facing early identification in the response phase. The context of interest included crisis hotlines and chat-based helplines, anti-violence support pathways, acute care and specialist services, legal support pathways, and digitally mediated first-contact environments when the stated purpose was to facilitate timely support, referral, reporting, or professional action.

The boundary of first-response support was defined explicitly. Eligible records had to show at least one of the following: direct survivor-facing first-line support; clinician-facing or advocate-facing decision support at first contact; service-adjacent triage, routing or reporting; or an enabling modality explicitly positioned for helplines, emergency care, specialist services, or support routing. We excluded records without an explicit AI component, records focused only on general awareness or prevention without a response pathway, studies not related to survivor support or early response, and secondary syntheses. Protocols and conceptual papers were retained only when they described an AI-enabled pathway model directly relevant to first-response workflows; they were interpreted separately from empirical evaluation studies.

### 2.4. Deduplication and Study Selection

Deduplication was performed deterministically, prioritising exact DOI matches followed by normalised title matching. Original title/abstract and full-text screening were conducted independently by two reviewers using predefined eligibility criteria, with discrepancies resolved by discussion and consensus. Supplementary IEEE/ACM screening and full-text decisions used the same criteria; they were undertaken by one reviewer, checked by a second reviewer, and resolved by final consensus. Screening decisions were recorded using a reason taxonomy to support transparent accounting of exclusions, particularly for records retained as background but not counted as included evidence.

### 2.5. Data Charting and Synthesis

Data charting used a structured extraction form capturing bibliographic information, document type, service setting, country or region, population and data source, AI approach, intended function, first-response relevance, evaluation design and outcomes, human oversight or escalation, and privacy, safety or equity considerations. The extraction form was piloted before full charting. Data charting was performed by one reviewer and subsequently checked by a second reviewer for completeness, consistency, and category assignment. Uncertainties or disagreements were resolved by final consensus. No study authors were contacted for additional information.

Findings were synthesised descriptively and organised into an evidence map spanning survivor-facing and hotline-adjacent digital support; first response in healthcare and specialist services; social-triage approaches for crisis services; legal/support-routing AI; and enabling modalities such as speech-based or privacy-oriented modelling. Given the scoping objectives and heterogeneity of study designs, quantitative pooling and formal risk-of-bias assessment were not undertaken.

To support structured interpretation of evaluative depth, each included source was assigned to an evidence maturity level based on the deepest evaluation reported: M0, technical development/validation only or protocol without evaluative results; M1, user-facing usability/acceptability evidence, systems audit, or qualitative implementation/ethics evidence; M2, workflow-integrated pilot testing with documented handover or escalation processes; and M3, pathway-level evaluation reporting service endpoints and safety monitoring. This author-developed rubric is a descriptive staging heuristic, not a validated critical appraisal instrument. It was informed by staged evaluation frameworks and evidence-tier approaches for digital health and AI systems [24,25,26]. Supplementary Table S5 provides a conceptual crosswalk with the Idea, Development, Exploration, Assessment and Long-term Study framework, the Developmental and Exploratory Clinical Investigations of Decision-support Systems Driven by Artificial Intelligence reporting guideline, and the National Institute for Health and Care Excellence Evidence Standards Framework; the crosswalk does not establish formal equivalence or validation. No independent validation or inter-rater reliability statistic was performed; therefore, the M0–M3 distribution should be interpreted as descriptive and potentially sensitive to boundary judgements.

## 3. Results

### 3.1. Search Results and Study Selection

The original database searches in Scopus, Web of Science Core Collection, and PubMed identified 187 records. After deterministic deduplication, 97 unique records were retained for title/abstract screening; 74 were excluded at this stage. Twenty-three reports were sought for retrieval; one report could not be retrieved. Twenty-two full texts were assessed for eligibility. One report was excluded because it was a systematic review and was used only for contextual discussion. The original search therefore contributed 21 included sources of evidence.

**Figure 1:**
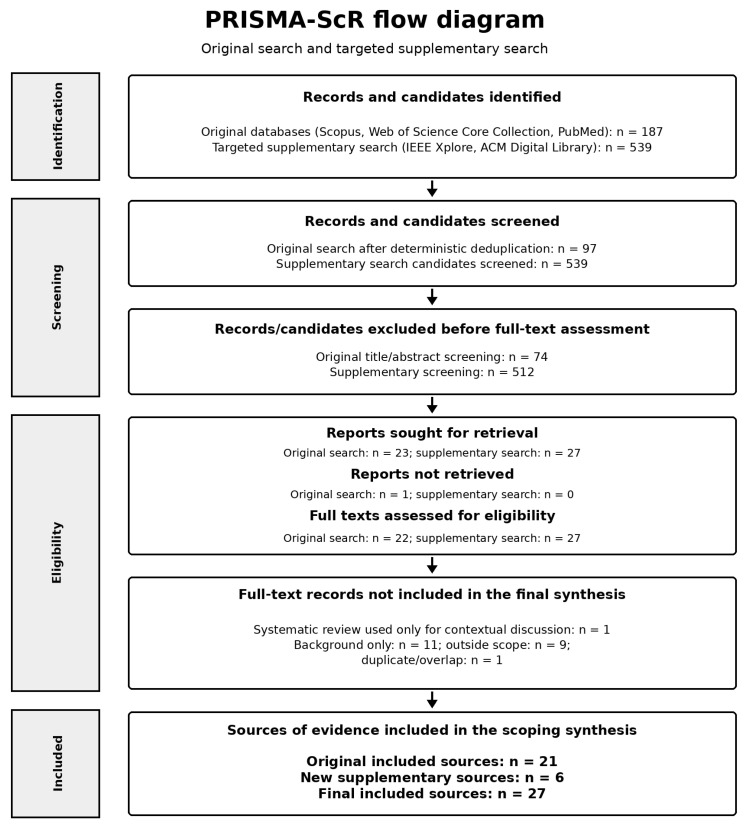
PRISMA-ScR flow diagram. Counts combine the original and targeted supplementary searches. ACM Digital Library results were screened in-platform; full search strategies, full-text screening decisions and final record accounting are provided in the Supplementary Materials.

The targeted supplementary search conducted on 9 July 2026 identified 123 IEEE Xplore records after within-source deduplication and 416 ACM Digital Library records/candidates screened in-platform. Residual overlap with the original search and between supplementary sources was resolved during screening. After title/abstract or in-platform screening, 512 supplementary records/candidates were excluded as out of scope, duplicate, secondary, or background only. Twenty-seven supplementary full texts were assessed. An amount of 6 additional sources met the eligibility criteria, yielding 27 sources of evidence in the final scoping synthesis (Figure 1).

### 3.2. Overview of Included Evidence

The 27 included sources of evidence (Table 1) represent a heterogeneous evidence base spanning survivor-facing first-line digital support, hotline-adjacent tools, first-response screening and triage in acute and specialist services, online environments framed as relevant to crisis routing, legal/support-routing tools, and enabling modalities such as speech-based modelling. Across the corpus, AI was predominantly presented as a supplementary tool for trained responders or support services, assisting information provision, screening, prioritisation, needs classification, legal guidance, reporting or routing, rather than replacing professional judgement, advocacy, clinical assessment or legal support [11,12,13,14,15,16,17,18,20,29,30,31,32,33,34,35,36,37,38,39,40,41,42,43,44,45,46].

Using the M0–M3 descriptive heuristic, the evidence remained concentrated at early maturity. Sixteen sources were categorised as M0, reporting technical development, retrospective validation, protocol-stage work, or conceptual workflow design without pathway testing. Eleven sources reached M1 through usability/acceptability evaluation, systems audit, preliminary user feedback, or qualitative implementation evidence. No included source reached M2 or M3: none reported a workflow-integrated pilot with documented handover or escalation processes, and none reported pathway-level service endpoints with safety or adverse-event monitoring. The resulting evidence maturity matrix is presented in Table 2.

The evidence base was geographically uneven. Sources and systems represented Europe, North America, East and South Asia, Southeast Asia, Africa, Latin America, Hong Kong, Kenya, Bangladesh, Thailand, India and Peru, as well as platform-based or multi-country chatbot audits. However, many records remained concentrated in high-resource digital-health or human–computer interaction settings, and several platform datasets were not anchored to a specific service jurisdiction. This limits generalisability across language, culture, legal systems, service capacity and digital safety conditions.

Of the 27 included sources, 25 reported empirical, technical, qualitative, usability, audit, design or evaluative data, 1 was a protocol, and 1 was a conceptual perspective retained because it directly addressed a first-response workflow. Detailed source characteristics and evaluation findings are provided in Supplementary Table S4a,b.

### 3.3. Survivor-Facing and Hotline-Adjacent First-Line Digital Support

The largest cluster addressed AI tools intended to support first-line contact with survivors via chatbots, hotline-integrated conversational agents, mobile applications, or digital help-seeking interfaces. Several records were explicitly survivor-facing. The AinoAid domestic violence chatbot was evaluated through a survivor-informed needs assessment and usability/acceptability work, including a large user survey; it was framed as a gateway to information and routing rather than as a replacement for professional support [11]. A 22-country audit of domestic violence chatbots identified deployed rule-based and LLM-powered tools and examined privacy policies, covertness, user interfaces, scenario responses and safety limitations [13]. Sexual violence chatbot studies included a hybrid rule-based/ML design study based on survivor-positioned questions [14] and a user-tested chatbot for image-based sexual abuse survivors that provided immediate information and emotional support [15].

Additional first-line digital tools included a Bangladesh domestic violence mobile application with an NLP chatbot, geolocation/emergency-alert action module and usability testing [16], and a Peruvian cloud platform with a conversational assistant for anonymous communication and specialist appointment routing in gender violence/discrimination contexts [17]. A mixed-methods study compared general-purpose LLM chatbots with a domain-specific domestic violence chatbot, focusing on response quality, empathy, bias and data responsibility [36]. A qualitative study with domestic violence survivors in Hong Kong examined preferred features of digital platforms and AI chatbots for help-seeking, emphasising safety, accessibility, stepwise guidance, non-judgemental feedback, and AI as complementary to human support [44].

Hotline-adjacent evidence also included qualitative and conceptual work on the boundary-setting problem that arises when services deploy or endorse LLM-powered chatbots. A document-based analysis of national sexual assault and domestic violence crisis hotlines examined how tools are positioned to callers and how communication strategies may shape expectations and anthropomorphisation risks [18]. Stakeholder interviews on domestic violence support chatbots highlighted duty of care, accountability, and explicit escalation pathways [20]. A conference perspective proposed AI-enabled domestic violence hotline screening, prioritisation, escalation and documentation, but did not report primary effectiveness evidence [33]. Prototype-oriented chatbots from Kenya and India reported technical testing or early user feedback without service-level outcomes [37,38]. Across this cluster, evaluation remained focused on usability, acceptability, audit findings, technical feasibility, or design assumptions rather than verified human handover, referral uptake, re-contact, or safety incidents.

### 3.4. First Response in Healthcare and Specialist Services

A second cluster focused on clinical and specialist first-contact settings, where NLP, ML and multimodal modelling were used to support identification, pre-screening, risk identification, or triage within clinician-led workflows. One large retrospective ED study applied rule-based NLP to clinical notes to identify IPV-related encounters beyond administrative coding, framing the tool as a mechanism to prompt screening and referral [29]. Another ED study used rule-based NLP on National Electronic Injury Surveillance System narratives to identify IPV-related visits and injury-context patterns [42]. A multimodal clinical model combined structured data and notes to identify IPV risk, reporting validation across cohorts while remaining a decision-support model rather than a tested service workflow [43].

Other healthcare and specialist-service studies included a supervised ML prescreening tool for probable traumatic brain injury among IPV patients [30], a Sexual Assault Referral Centre triage model for pre-existing mental health difficulties in child sexual assault cases [31], and a protocol for AI-enabled modelling of psychosocial, judicial and post-traumatic stress disorder trajectories after a recent sexual assault in a forensic medical centre pathway [32]. Across these studies, AI was framed as a screening, surveillance or flagging layer embedded within human-led clinical assessment and referral. Human oversight was therefore implicit in the setting, but escalation triggers, workflow behaviour, service endpoints and safety monitoring were generally not operationalised.

### 3.5. Social-Triage Approaches for Crisis Services

Five studies applied ML, deep learning, LLMs or topic modelling to social media, online community, or online forum data with the explicit or implied rationale of supporting prioritisation, timely response, or professional interpretation of survivor disclosures. Early Facebook-based domestic violence studies classified critical posts, post types, or disclosure intent to support crisis attention or routing [34,39,40]. An LLM-based classifier categorised survivors’ information needs from online health community posts into categories such as legal support, safety planning and shelters; external validation showed performance decline, underscoring domain shift and portability limitations [35]. A mixed qualitative and ML study of IPV forum posts used supervised classifiers and topic modelling to identify IPV subtypes and contextual patterns [45]. These records reported model performance or analytic insight from labelled/coded corpora but rarely demonstrated live integration into staffed services or verified downstream outcomes.

### 3.6. Legal/Support-Routing AI

Two included studies focused primarily on legal or support routing. LAW-U is a Thai AI chatbot that provides legal guidance to sexual violence survivors by matching user-described incidents to relevant Supreme Court decisions; it reported model-development and technical-validation results but no deployed service outcome evaluation [12]. A later Indian retrieval-augmented legal companion, NyayaSakhi-SWATI, provided plain-language legal literacy, likely statutory relief prediction and next-step recommendations under domestic violence law; it reported prototype/model evaluation and preliminary survivor/practitioner perceptions but no healthcare or pathway-level outcome evaluation [46]. These records indicate a potentially important legal support direction, but they should not be interpreted as validated healthcare or crisis-response interventions.

### 3.7. Enabling Modalities and Privacy-Oriented Modelling

One technical study explored speech-based identification of a GBV-related victim condition and investigated speaker-agnostic modelling to reduce re-identification risk, proposing conceptual applicability to helpline and telehealth contexts [41]. While aligned with the privacy-sensitive nature of first-response contexts, this work remains at the model-development stage and does not demonstrate service integration or pathway-level outcomes.

### 3.8. Outcomes, Service Endpoints and Digital Trace Safety

Reported outcomes clustered into technical performance, usability/acceptability, systems audit, and qualitative implementation or governance findings. Technical performance metrics such as accuracy, harmonic-mean precision–recall scores, area under the receiver operating characteristic curve, precision, recall and response time predominated in healthcare screening, social-triage, legal natural language processing, needs classification and speech-based studies [12,14,29,30,31,32,33,34,35,37,39,40,41,42,43,45]. Usability, acceptability, interface evaluation or user feedback appeared mainly in survivor-facing chatbots, mobile applications and digital support tools [11,13,15,16,17,18,36,38,44,46]. Qualitative governance findings addressed boundary-setting, accountability and implementation conditions [18,20,33].

No included source reported a complete workflow-integrated evaluation of human escalation, referral completion or pathway-level safety. Isolated pathway-relevant elements were described or audited, but not prospectively evaluated. Digital trace safety was addressed more often in the broader corpus after the targeted supplementary search, but systematic measurement remained limited. Safe-exit functionality, data deletion, no-history modes, device monitoring risk, abuser access, privacy-preserving retention, multilingual/cultural safety and incident response were rarely evaluated as outcomes. When addressed, they were more often design considerations or audit findings than tested implementation endpoints [13,15,20,41].

## 4. Discussion

### 4.1. Pathway-Centric Interpretation and Evidence Maturity

This scoping review indicates that AI in violence response is not a single application class. It is a set of partially overlapping functions distributed across survivor-facing chatbots, mobile reporting and support tools, hotline-adjacent conversational systems, clinical screening and surveillance models, online disclosure classifiers, legal/support-routing companions, and enabling modalities such as speech or privacy-preserving modelling. The dominant logic remains augmentation: AI is positioned as a way to standardise intake, accelerate triage, provide bounded information, or support routing to human services rather than replacing professional judgement [33].

The literature’s centre of gravity remains upstream of demonstrable service impact. Even when systems are explicitly designed for survivor-facing contexts, evaluation commonly stops at technical performance, usability, acceptability, audit findings or early prototype feedback. The targeted IEEE Xplore and ACM Digital Library searches broadened the map of survivor-facing chatbots and digital tools, but did not alter the maturity pattern: no source reached M2 or M3.

This interpretation is consistent with a recent systematic review on AI for IPV prevention, which identified promising applications across ML, NLP, image analysis and chatbots while emphasising persistent gaps in long-term effectiveness, equity, privacy, safety and implementation across health and social care systems [47]. The present first-response mapping therefore supports a cautious reading: feasibility and performance signals are accumulating faster than evidence on survivor-centred outcomes, escalation quality, adverse events, or sustained service integration.

At the same time, a pathway-centred interpretation should not reduce the first response to classification and transfer. The current corpus does not support firm conclusions about relational quality, but survivor-centred and hotline research indicates that disclosure is often hesitant, emotionally demanding and dependent on control over timing, wording and depth [48,49,50,51]. An AI-supported pathway may therefore appear efficient while still failing the survivor if it prompts premature disclosure, increases avoidable re-telling or turns listening into data capture.

The value of a low-threshold digital gateway lies not only in what it accelerates, but also in what it protects: survivor agency and a safe transition to accountable human support. Future evaluations should therefore ask whether these systems leave room for recognition, witnessing, listening and care [49,50,51,52]. These considerations are interpretive safeguards, not outcomes demonstrated by the present evidence base.

### 4.2. Managing Heterogeneity and Scope

The explicit eligibility boundary helps interpret heterogeneity without treating all records as equivalent. Direct survivor-facing tools, clinician-facing early identification systems, service-adjacent social-triage classifiers, legal support companions and enabling speech models were mapped together because each was explicitly positioned near early support, reporting, triage or routing. However, their evidentiary implications differ. The mapped evidence also spans child sexual assault, adult sexual violence, intimate partner violence, domestic violence and broader gender-based violence; these involve distinct clinical, legal, cultural and service contexts and should not be read as interchangeable across populations or pathways. A retrospective ED natural language processing model, a deployed chatbot audit, a legal-advice chatbot, and a prototype mobile application do not support the same claim. The Results and Tables, therefore, separate document type, first-response relevance, evidence type and maturity level. Protocols, conceptual papers and technical feasibility records should be read as map entries, not as evidence of service effectiveness.

### 4.3. Safety-Critical Escalation and Handover Requirements

Across the included evidence, escalation to human support is widely invoked but inconsistently specified. Hotline-facing and chatbot-audit studies underscore that users may infer capacities that exceed the tool’s intended scope, making boundary setting and expectation management part of safety [13,18]. Stakeholder-oriented work similarly argues that governance must include clear lines of responsibility and explicit escalation pathways [20]. Yet no included source operationalised escalation as a testable workflow element with defined triggers, time constraints, fallback behaviour and failure handling.

On this basis, Table 3 offers a set of author-informed minimum safety considerations for survivor-facing first-response AI. The table is not intended to function as a guideline. It brings together the review findings on escalation and boundary setting with adjacent work on domestic abuse chatbot design, sexual violence conversational agents and text-based hotline interaction [48,49,50]. It also reflects survivor-facing chatbot and humanitarian GBV analyses, which emphasise access, handoff and the need to avoid substituting automated interaction for human support [51,52]. More general work on conversational agents is used more narrowly, to caution against untested claims about simulated empathy, anthropomorphism and mental health safety [53,54,55].

### 4.4. What to Measure: Core Outcomes and Evaluation Questions

Technical performance dominates evaluation across multiple clusters. This creates an interpretive imbalance: it is currently easier to claim success for documentation, detection or classification tasks than for survivor-facing first-line support. Future pilot studies should treat service integration as an explicit object of evaluation rather than inferring pathway value from model accuracy alone. The distinction between survivor-centred outcomes and service outcomes is critical. Survivor-centred outcomes may include perceived safety, trust, emotional distress, empowerment, control over disclosure, reduced re-traumatisation and successful connection to chosen support. Service outcomes may include escalation accuracy, handover completion, referral uptake, response time, responder workload, misrouting and adverse-event reporting.

### 4.5. Equity, Adversarial Context, Privacy, and Governance

Equity is often discussed in general terms, but the included sources point to more concrete failure modes. Models trained on narrow linguistic, clinical or platform-specific data can degrade when applied to different populations, languages and service contexts. Domestic violence chatbot ethics work and large-language-model-based needs classification show how these issues arise within GBV-related support contexts [20,35]. The adjacent AI and violence literature helps frame such failures as ethical and survivor-safety concerns, not only as decreases in technical performance [56,57]. The privacy-preserving AI literature supports data minimisation and trace-aware design [58], while broader work on fairness, inclusivity and intersectional AI supports subgroup and cross-context reporting [59,60,61]. Decolonial and privacy-preserving perspectives also caution against assuming that a tool trained or validated in one setting will be safe, acceptable or portable in another [62,63].

Violence response also operates in an adversarial environment. Device monitoring, shared accounts, compelled disclosure, coercive control and retaliation create safety risks that differ from typical healthcare chatbot deployments. Deployed chatbot audits identified gaps in privacy policies and covertness [13], and experimental digital safety research shows how conversational AI may be weaponised to facilitate, exacerbate or commoditise coercive control [64]. For first-response AI, trace exposure, access compromise, behavioural coercion and unintended disclosure should be treated as baseline design conditions, not edge cases. Box 1 summarises an illustrative threat model.

Box 1 **Threat model for AI-enabled first response under coercive control and constrained digital autonomy.**

**Key risk conditions to assume as baseline, not exceptions**

**Access compromise**
Perpetrators may monitor devices, shared accounts, call logs, browser history, or messaging apps, turning routine interaction traces into direct safety risks.

**Forced disclosure and retaliation**
Survivors may be compelled to reveal interactions or may face retaliation triggered by notifications, recommendations, referral prompts, or visible digital traces left by the system.

**Model manipulation and content steering**
Survivor-facing LLM systems may be exposed to prompt manipulation, indirect prompt injection through pasted text, or adversarial elicitation of unsafe guidance.

**Trace amplification through logging and analytics**
Back-end telemetry, conversation storage, and model-improvement pipelines may create persistent sensitive artefacts even when the user-facing interface appears ephemeral.

**Service denial and overload**
False-positive escalation, malicious misuse, or adversarial triggering may saturate human responders, degrading availability precisely for high-risk users.

Governance cannot remain abstract where AI is positioned within first-response workflows. Service-integrated AI requires clearer specification of risk ownership, incident response, auditability, privacy-minimising logging, drift monitoring, and procedures for misrouting, failed escalation or unsafe outputs. The currently mapped evidence remains too limited to support strong conclusions about how these governance arrangements should be standardised across settings. It supports staged, cautious evaluation rather than immediate operational normalisation.

### 4.6. Strengths and Limitations

This review focuses specifically on AI in first-response and victim-service contexts and distinguishes service-adjacent applications from broader AI-for-health or AI-for-violence-prevention literature. The targeted supplementary search in IEEE Xplore and ACM Digital Library strengthened computer-science and human–computer interaction coverage and added relevant survivor-facing chatbot, legal support and digital-reporting studies. The review also provides explicit first-response categories, a shortened main table, detailed supplementary extraction matrices, a conceptual crosswalk for the M0–M3 heuristic, and transparent supplementary full-text screening decisions.

Limitations remain. First, no review protocol was prospectively registered, and no retrospective registration was undertaken or is planned. Second, although retrieval did not initially apply language filters, eligibility was limited to English-language full texts, introducing potential language bias. Third, the database set still excluded grey literature, regional databases, social work and criminology databases, and operational documentation from hotline or anti-violence organisations. This likely underrepresents real-world tools, local implementation reports and service practice in low- and middle-income countries. Fourth, the evidence base is heterogeneous and early-stage, mixing technical validation, usability studies, qualitative implementation work, protocols and conceptual pathway proposals. These records should not be interpreted as equivalent evidence of effectiveness.

Fifth, the M0–M3 maturity rubric is author-developed and was applied by the same team. It is useful as a descriptive heuristic, but it is not a validated quality-appraisal tool, was not independently calibrated, and was not accompanied by a kappa statistic or independent inter-rater reliability test. The classification should therefore be read as transparent evidence staging rather than evaluative authority. Sixth, funding, commercial involvement and product status of primary tools were charted where available but not formally appraised. Named products, including AinoAid and other chatbots, are discussed descriptively and without endorsement. Finally, the concentration of M0 evidence may reflect both the early stage of the field and publication patterns that favour technical development and feasibility work over negative or service-integrated evaluations.

The included sources indicate momentum towards AI-enabled intake, triage, reporting, legal guidance and navigation in violence-response pathways, but they also expose a consistent translational gap between technical feasibility and service-integrated safety and impact. The author-informed considerations presented in Table 3 and Table 4, Box 1 and Section 5 are intended to support future pilot and implementation work; they are not formally validated framework outputs. These proposals require further refinement through survivor and stakeholder involvement, formal consensus processes where appropriate, and prospective pilot and implementation testing.

## 5. Conclusions

This scoping review maps a rapidly developing yet still uneven body of peer-reviewed evidence on AI-enabled tools intended to support early-stage assistance for survivors of sexual violence and GBV across first-response pathways. After targeted supplementary searching in IEEE Xplore and ACM Digital Library, the final corpus comprised 27 included sources of evidence: 25 empirical, technical, qualitative or evaluative records, 1 protocol and 1 conceptual perspective. The mapped literature concentrates on practical first-contact functions such as information support, screening, surveillance, prioritisation, needs classification, reporting, legal guidance, risk identification and routing. However, the evidentiary pattern remains dominated by feasibility demonstrations, technical validation, systems audits, qualitative acceptability work and prototype evaluation rather than by verified service-integrated effectiveness or safety outcomes.

The review, therefore, supports a cautious, pathway-aware interpretation of the field. Across settings, intended functions and model-level performance are reported more often than downstream evidence on escalation quality, referral uptake, re-contact patterns, adverse events, survivor-centred outcomes or other pathway-level endpoints. Future studies should report pathway relevance more explicitly and strengthen the evaluation of human escalation, external validity, equity, privacy, digital trace safety, and downstream service outcomes. The practice-oriented considerations outlined here should be read as author-informed proposals for future pilot and implementation work, not as formally validated guidelines.

## Figures and Tables

**Table 1:** Short characteristics of included sources of evidence (*n* = 27). Detailed extraction matrices are provided in Supplementary Table S4a,b.

Source	Setting/Pathway Point	First-Response Relevance	AI Component/ Function	Evidence Type	Maturity
Vogt et al. [11], 2026	Domestic violence support chatbot; Europe/Germany	Direct survivor-facing first-line support	Domain-specific chatbot with structured dialogue/NLP	Mixed-methods needs and user evaluation	M1
Socatiyanurak et al. [12], 2021	Sexual violence legal guidance; Thailand	Survivor-facing legal/support routing	AI chatbot using NLP/text-similarity over Supreme Court cases	Model development and technical validation	M0
Poveda et al. [13], 2026	Deployed domestic violence chatbots; 22-country audit	Survivor-facing chatbot ecosystem and safety audit	Rule-based and LLM-powered domestic violence chatbots	Systems audit, user-interface/user-experience and scenario-based evaluation	M1
Maeng and Lee [14], 2021	Sexual violence support chatbot design; Korea	Survivor-facing conversational support design	Hybrid rule-based and ML chatbot concept	Exploratory question analysis and technical design study	M0
Maeng and Lee [15], 2022	Image-based sexual abuse support; Korea	Direct first-contact information and emotional support	Chatbot for image-based sexual abuse survivors	Prototype development and user study	M1
Hossain et al. [16], 2020	Domestic violence mobile app; Bangladesh	Digital first-contact support, emergency alert and information	Mobile app with NLP chatbot and action module	Prototype testing and system usability scale evaluation	M1
Arrascue Casara et al. [17], 2023	Gender violence/discrimination support; Peru	Anonymous support and appointment routing	Cloud platform with conversational AI/virtual assistant	Prototype validation with women and specialists	M1
Wise [18], 2025	National sexual assault and domestic violence hotlines	Hotline-adjacent chatbot boundary setting	LLM-powered chatbots as described by services	Qualitative service-documentation study	M1
Mielismäki and Husso [20], 2025	Domestic violence support services; ethics/governance	Implementation conditions for domestic violence chatbots	AI-driven chatbots, technology-agnostic	Qualitative stakeholder study	M1
Tabaie et al. [29], 2022	Emergency department electronic health record notes; United States	Clinician-facing first-contact identification	Rule-based NLP	Retrospective observational study with technical validation	M0
Sachdeva et al. [30], 2024	ED/IPV traumatic brain injury prescreening	Clinician-facing prescreening	Supervised ML ensemble on clinical reports	Model development and internal validation	M0
Majeed-Ariss et al. [31], 2025	Sexual Assault Referral Centre; United Kingdom	Specialist-service triage at first contact	Prediction model/logistic regression	Model development with internal validation	M0
Fedele et al. [32], 2023	Forensic medical centre after recent sexual assault; France	Planned pathway modelling and follow-up tailoring	Planned predictive algorithms and trajectory modelling	Protocol; no results	M0
Yazıcıoğlu and Yalçın Sarıbey [33], 2025	Domestic violence hotline workflow proposal	Hotline screening, prioritisation and documentation	NLP, speech sentiment and predictive analytics proposed	Conceptual perspective; no primary evaluation	M0
Subramani et al. [34], 2018	Domestic violence Facebook posts	Social-triage prioritisation for crisis services	Deep-learning text classification	Technical feasibility study	M0
Guan et al. [35], 2025	Online health community posts	Professional decision support for needs triage	Fine-tuned GPT-3.5/LLM classifier	Model development with external validation	M0
Sanz Urquijo et al. [36], 2025	GBV chatbot response evaluation; Spain	First-line digital support response quality	General-purpose LLMs and domain-specific chatbots	Mixed-methods survivor-informed comparative evaluation	M1
Mutinda and Muchiri [37], 2024	GBV counselling chatbot; Kenya	First-contact information and referral	Rasa intent classification and dialogue management	Technical feasibility study	M0
Awasekar and Lobo [38], 2025	Domestic violence awareness/legal chatbot; India	Survivor-facing information and service navigation	NLP/rule-based chatbot	Prototype description with preliminary user feedback	M1
Subramani et al. [39], 2019	Domestic violence online posts/support groups	Social-triage need categorisation	Deep-learning multi-class classification	Technical feasibility study	M0
Subramani et al. [40], 2017	Domestic violence discourse on social media	Online disclosure/intent classification	Classical ML with feature engineering	Technical feasibility study	M0
Reyner-Fuentes et al. [41], 2025	Speech-based GBV screening research	Enabling modality for helplines/telehealth	Domain-adversarial speaker-agnostic modelling	Model development and validation	M0
Barboza-Salerno et al. [42], 2026	Emergency department narrative surveillance; United States	ED surveillance/screening support	Rule-based NLP over National Electronic Injury Surveillance System narratives	Retrospective observational NLP surveillance	M0
Gu et al. [43], 2026	Clinical IPV risk identification; United States	Clinician decision support before/help-seeking	Multimodal ML using notes and structured data	Model development and validation	M0
Hui et al. [44], 2026	Digital platform/chatbot preferences; Hong Kong	Survivor-informed design for help-seeking	Qualitative study of AI chatbot/digital platform features	Qualitative survivor-perspectives study	M1
Zhang et al. [45], 2026	IPV online forums	Analytic support for identifying disclosure patterns	Qualitative coding, random forest, and neural-network classifiers and topic modelling	Mixed qualitative/ML analytic study	M0
Awasekar and Lobo [46], 2026	Legal/support routing AI companion; India	Survivor-facing legal/support routing	Retrieval-augmented LAMP2 4.0/SWATI interface	Prototype/model evaluation with preliminary feedback	M1

**Table 2:** Evidence maturity matrix. M0–M3 is an author-developed descriptive heuristic, not a validated quality-appraisal tool.

Domain	M0	M1	M2	M3
Hotlines and survivor-facing first-line digital support	[14,33,37]	[11,13,15,16,17,18,20,36,38,44]	-	-
Healthcare and specialist services	[29,30,31,32,42,43]	-	-	-
Social-triage and online environments	[34,35,39,40,45]	-	-	-
Enabling modalities (e.g., speech/privacy)	[41]	-	-	-
Legal/support-routing AI companion	[12]	[46]	-	-

**Table 3:** Author-informed minimum safety considerations for survivor-facing first-response AI.

Element	Minimum Consideration	Evidence to Report
Escalation triggers	Predefined red-flag signals that prompt handover to a trained human responder.	Trigger list, threshold logic, sensitivity analyses, and false-positive/false-negative handling.
Time-bounded handover	Rules for maximum time in automated mode and behaviour when no human is available.	Response-time targets, after-hours fallback, and documented failure modes.
Structured handoff summary	Survivor-controlled summary to reduce re-telling and preserve trauma-informed interaction.	Handoff template, consent controls, completeness and user-control measures.
Boundary setting and transparency	Clear statements of capabilities, limits and emergency guidance; avoid misleading anthropomorphism.	Disclosure text, comprehension checks, usability or audit findings.
Privacy minimisation and trace safety	Default minimisation of stored data and on-device traces.	Retention policy, data-flow summary, quick-exit/no-history options, coercive-control risk assessment.
Audit and accountability	Decision trail sufficient for incident review while respecting privacy minimisation.	Audit fields, access controls, governance owner and role allocation.
Safety incident response	Process for detecting, reporting and remediating unsafe outputs or escalation failures.	Incident taxonomy, monitoring plan, remediation timelines and post-incident review.
Equity and portability	External validation and subgroup reporting for tools that route or prioritise survivors.	External datasets, subgroup performance, language and cultural portability limits.

**Table 4:** Candidate outcome domains for future pilot evaluation. These are author-informed proposals, not a consensus-derived core outcome set.

Domain	Core Outcome	Example Operationalisation
Service performance	Time-to-human escalation	Median time from first contact to successful handover; stratify by service hours and trigger type.
Service performance	Successful routing rate	Contacts routed to an appropriate service pathway as verified by follow-up or logs.
Service performance	Drop-off during intake	Users disengaging before completing minimum intake or safety screening; report stage.
Service performance	Responder workload	Queue length, call/chat duration, documentation time and perceived workload.
Survivor-centred outcomes	Perceived safety and control	Post-contact safety perception, control over disclosure depth, trust and clarity.
Survivor-centred outcomes	Distress and re-traumatisation	Emotional distress, perceived empathy, avoidance of coercive or premature disclosure.
Safety	Escalation failure	Red-flag content that does not trigger or complete escalation within the specified time.
Safety	Unsafe content incidents	Advice that increases risk, encourages unsafe disclosure or contradicts service protocols.
Equity/portability	Subgroup performance deltas	Performance across language, demographic or accessibility strata; report missing data.
Digital safety/privacy	Trace footprint	Retention, storage, deletion, quick-exit/no-history features and data-flow summary.
Digital safety/privacy	Compromise risk assessment	Mitigations for device monitoring, shared accounts and coercive-control scenarios.

## Data Availability

No new data were created or analysed in this study. Data sharing is not applicable to this article.

## Supplementary Materials

The following supporting information can be downloaded at: https://www.mdpi.com/article/10.3390/healthcare14142174/s1. Section S1. Search strategy and targeted supplementary search summary. Table S1. Original database-specific search strategies. Table S2. Targeted supplementary search strategies (9 July 2026). Table S3. Targeted supplementary search summary. Section S2. Complete PRISMA-ScR checklist. Section S3. Data charting form. Section S4. Detailed extraction matrices. Table S4. (a). Source characteristics and first-response relevance. (b). Evaluation, implementation, safety and evidence interpretation. Section S5. Conceptual crosswalk for the M0–M3 descriptive heuristic. Table S5. Conceptual crosswalk between the author-developed M0–M3 heuristic and established staged-evaluation frameworks. Section S6. Source-level M0–M3 coding matrix. Table S6. Source-level M0–M3 coding matrix. Section S7. Supplementary full-text screening decisions. Table S7. Supplementary full-text screening decisions. Section S8. Final search record accounting. Table S8. Final search record accounting.

## Abbreviations

The following abbreviations are used in this manuscript:

AI	artificial intelligence
ED	emergency department
GBV	gender-based violence
IPV	intimate partner violence
LLM	large language model
ML	machine learning
NLP	natural language processing
