# Supplementary material for: AI-Enabled First-Response Support After Sexual and Gender-Based Violence: A PRISMA-ScR Scoping Review
Source: Healthcare (Basel). 2026 Jul 18;14(14):2174. doi: 10.3390/healthcare14142174 (PMC13410215; doi:10.3390/healthcare14142174)
Supplement: Supplementary file 1 [file healthcare-14-02174-s001.zip › healthcare-4418297-supplementary.pdf]

Supplementary Materials

Search strategy, targeted-search summary, complete PRISMA-ScR checklist, data charting form, detailed extraction matrices, conceptual crosswalk, source-level coding matrix, full-text screening decisions, and final record accounting

AI-Enabled First-Response Support After Sexual and Gender-Based Violence: A PRISMA-ScR Scoping Review

This supplement documents the original database searches completed on 16 June 2026 and the targeted supplementary IEEE Xplore/ACM Digital Library search completed on 9 July 2026. The final evidence map includes 27 sources of evidence. The M0-M3 levels are descriptive staging categories and are not a formal critical appraisal.

Abbreviations: ACM, Association for Computing Machinery; AI, artificial intelligence; DECIDE-AI, Developmental and Exploratory Clinical Investigations of Decision-support systems driven by Artificial Intelligence; ED, emergency department; EHR, electronic health record; GBV, gender-based violence; IDEAL, Idea, Development, Exploration, Assessment and Long-term study; IEEE, Institute of Electrical and Electronics Engineers; IPV, intimate partner violence; LLM, large language model; M0-M3, author-developed evidence maturity levels; ML, machine learning; NEISS, National Electronic Injury Surveillance System; NICE ESF, National Institute for Health and Care Excellence Evidence Standards Framework; NLP, natural language processing; PRISMA-ScR, Preferred Reporting Items for Systematic Reviews and Meta-Analyses extension for Scoping Reviews; PTSD, post-traumatic stress disorder; RAG, retrieval-augmented generation; SARC, Sexual Assault Referral Centre.

Section S1. Search strategy and targeted supplementary search summary

The search combined three concept blocks: sexual violence/gender-based violence; first-response or victim-service contexts; and artificial intelligence methods/systems. No language filter was applied at retrieval. Eligibility was subsequently limited to English-language full texts. IEEE Xplore records were deduplicated within source. ACM Digital Library results were screened in-platform because bulk structured export was unavailable. Potentially eligible records were retained for full-text assessment, and the corresponding full-text decisions are reported in Section S7. Residual overlaps between supplementary sources and with the original corpus were resolved during screening.

Table S1. Original database-specific search strategies.

Source/strategy	Search string
Scopus broad	TITLE-ABS-KEY(("sexual assault" OR rape OR "sex offense" OR "sexual violence" OR "gender based violence" OR GBV OR "intimate partner violence" OR IPV OR "domestic violence") AND ("crisis hotline" OR hotline OR helpline OR "crisis line" OR "rape crisis" OR "sexual assault referral" OR SARC OR "forensic medical" OR "forensic medical center" OR "emergency department" OR "anti violence" OR "victim service" OR advocacy OR shelter OR counselling OR "online community" OR "online health community" OR "social media") AND ("artificial intelligence" OR "machine learning" OR "deep learning" OR "natural language processing" OR NLP OR "large language model" OR "large language models" OR LLM OR LLMs OR chatbot OR chatbots OR "conversational agent" OR "conversational agents" OR "speech recognition" OR "sentiment analysis")) AND NOT DOCTYPE(re)
Scopus narrow	TITLE-ABS-KEY(("sexual assault" OR rape OR "sex offense" OR "sexual violence" OR "gender based violence" OR GBV OR "intimate partner violence" OR IPV OR "domestic violence") AND ("crisis hotline" OR hotline OR helpline OR "rape crisis" OR "sexual assault referral centre" OR "sexual assault referral center" OR SARC OR "anti violence center" OR "anti violence centre") AND ("artificial intelligence" OR "machine learning" OR "deep learning" OR "natural language processing" OR NLP OR "large language model" OR "large language models" OR LLM OR LLMs OR chatbot OR chatbots OR "conversational agent" OR "conversational agents" OR "speech recognition" OR "sentiment analysis")) AND NOT DOCTYPE(re)
Web of Science broad	TS=(("sexual assault" OR rape OR "sex offense" OR "sexual violence" OR "gender based violence" OR GBV OR "intimate partner violence" OR IPV OR "domestic violence") AND ("crisis hotline" OR hotline OR helpline OR "crisis line" OR "rape crisis" OR "sexual assault referral" OR SARC OR "forensic medical" OR "forensic medical center" OR "emergency department" OR "anti violence" OR "victim service" OR advocacy OR shelter OR counselling OR "online community" OR "online health community" OR "social media") AND ("artificial intelligence" OR "machine learning" OR "deep learning" OR "natural language processing" OR NLP OR "large language model" OR "large language models" OR LLM OR LLMs OR chatbot OR chatbots OR "conversational agent" OR "conversational agents" OR "speech recognition" OR "sentiment analysis")) NOT DT=(Review)
Web of Science narrow	TS=(("sexual assault" OR rape OR "sex offense" OR "sexual violence" OR "gender based violence" OR GBV OR "intimate partner violence" OR IPV OR "domestic violence") AND ("crisis hotline" OR hotline OR helpline OR "rape crisis" OR "sexual assault referral centre" OR "sexual assault referral center" OR SARC OR "anti violence center" OR "anti violence centre") AND ("artificial intelligence" OR "machine learning" OR "deep learning" OR "natural language processing" OR NLP OR "large language model" OR "large language models" OR LLM OR LLMs OR chatbot OR chatbots OR "conversational agent" OR "conversational agents" OR "speech recognition" OR "sentiment analysis")) NOT DT=(Review)
PubMed broad	(("Sex Offenses"[MeSH] OR "Rape"[MeSH] OR "Domestic Violence"[MeSH] OR "Intimate Partner Violence"[MeSH] OR "sexual assault"[Title/Abstract] OR rape[Title/Abstract] OR "sexual violence"[Title/Abstract] OR "gender based violence"[Title/Abstract] OR GBV[Title/Abstract] OR IPV[Title/Abstract] OR "domestic violence"[Title/Abstract]) AND ("crisis hotline"[Title/Abstract] OR hotline[Title/Abstract] OR helpline[Title/Abstract] OR "crisis line"[Title/Abstract] OR "rape crisis"[Title/Abstract] OR "sexual assault referral"[Title/Abstract] OR SARC[Title/Abstract] OR "emergency department"[Title/Abstract] OR "forensic medical"[Title/Abstract] OR "victim service"[Title/Abstract] OR advocacy[Title/Abstract] OR shelter[Title/Abstract] OR counselling[Title/Abstract] OR "online health community"[Title/Abstract] OR "social media"[Title/Abstract]) AND ("artificial intelligence"[Title/Abstract] OR "machine learning"[Title/Abstract] OR "deep learning"[Title/Abstract] OR "natural language processing"[Title/Abstract] OR NLP[Title/Abstract] OR "large language model"[Title/Abstract] OR LLM[Title/Abstract] OR chatbot[Title/Abstract] OR "conversational agent"[Title/Abstract] OR "speech recognition"[Title/Abstract] OR "sentiment analysis"[Title/Abstract])) NOT (review[Publication Type] OR systematic[Title] OR meta analysis[Title])
PubMed narrow	(("Sex Offenses"[MeSH] OR "Rape"[MeSH] OR "Domestic Violence"[MeSH] OR "Intimate Partner Violence"[MeSH] OR "sexual assault"[Title/Abstract] OR rape[Title/Abstract] OR "sexual violence"[Title/Abstract] OR "gender based violence"[Title/Abstract] OR GBV[Title/Abstract] OR IPV[Title/Abstract] OR "domestic violence"[Title/Abstract]) AND ("crisis hotline"[Title/Abstract] OR hotline[Title/Abstract] OR helpline[Title/Abstract] OR "rape crisis"[Title/Abstract] OR "sexual assault referral centre"[Title/Abstract] OR "sexual assault referral center"[Title/Abstract] OR SARC[Title/Abstract]) AND ("artificial intelligence"[Title/Abstract] OR "machine learning"[Title/Abstract] OR "natural language processing"[Title/Abstract] OR NLP[Title/Abstract] OR "large language model"[Title/Abstract] OR LLM[Title/Abstract] OR chatbot[Title/Abstract])) NOT (review[Publication Type] OR systematic[Title] OR meta analysis[Title])

Table S2. Targeted supplementary search strategies (9 July 2026).

Source/strategy	Search string or query set
IEEE Xplore principal	("sexual assault" OR rape OR "sexual violence" OR "gender-based violence" OR "gender based violence" OR GBV OR "intimate partner violence" OR IPV OR "domestic violence" OR "domestic abuse" OR "violence against women") AND (hotline OR helpline OR "crisis line" OR "rape crisis" OR "victim support" OR "victim service" OR shelter OR advocacy OR counselling OR counseling OR "safety planning" OR "emergency department" OR "online community" OR "social media" OR "digital intervention" OR "technology-facilitated abuse") AND ("artificial intelligence" OR "machine learning" OR "deep learning" OR "natural language processing" OR NLP OR "large language model" OR LLM OR chatbot OR "conversational agent" OR "speech recognition" OR "sentiment analysis")
IEEE Xplore narrow	("sexual assault" OR "sexual violence" OR "gender-based violence" OR "gender based violence" OR "intimate partner violence" OR "domestic violence" OR "domestic abuse") AND (hotline OR helpline OR chatbot OR "conversational agent" OR "victim support" OR "safety planning" OR "social media" OR "online community") AND ("artificial intelligence" OR "machine learning" OR "deep learning" OR "natural language processing" OR NLP OR LLM OR "large language model")
ACM Digital Library manual queries	"domestic violence" chatbot; "domestic violence" "conversational agent"; "domestic violence" "machine learning"; "domestic violence" "natural language processing"; "intimate partner violence" "machine learning"; "intimate partner violence" "natural language processing"; "gender-based violence" chatbot; "gender based violence" chatbot; "sexual assault" chatbot; "sexual assault" "natural language processing"; "violence against women" "machine learning"; "safety planning" chatbot "domestic violence"; "victim support" chatbot "domestic violence".

Table S3. Targeted supplementary search summary.

Source	Strategy	Record handling	Count	Screening note
IEEE Xplore	Principal and narrow strategies	Structured comma-separated values export	104 principal; 96 narrow; 123 records after within-source deduplication	Title/abstract screening followed by full-text assessment using the same eligibility criteria
ACM Digital Library	Predefined keyword-query set	In-platform screening; potentially eligible records retained for full-text assessment	416 records/candidates screened in-platform	Bulk export unavailable through the accessed interface; full-text decisions are reported in Section S7
Combined supplementary stream	IEEE + ACM	Within-source deduplication/screening; residual cross-source/original overlaps resolved during screening	539 records/candidates screened; 27 full texts assessed; 6 included	512 excluded before full-text assessment; full-text reasons reported in Section S7

Section S2. Complete PRISMA-ScR checklist

Page references refer to the accompanying main manuscript. "Not applicable" is used only where the review did not undertake the optional procedure.

Item	Requirement	Status	Location	Note
1	Identify the report as a scoping review.	Reported	p. 1, Title	Identifies the report as a scoping review.
2	Provide a structured summary that includes, as applicable: background, objectives, eligibility criteria, sources of evidence, charting methods, results, and conclusions related to the review questions and objectives.	Reported	p. 1, Abstract	Background, methods, sources, selection, results and conclusions are summarised.
3	Describe the rationale for the review in the context of what is already known. Explain why the review questions/objectives lend themselves to a scoping review approach.	Reported	pp. 2-3, Introduction	Rationale and the need for evidence mapping are described.
4	Provide an explicit statement of the questions and objectives being addressed, with reference to their key elements (e.g., population, concepts, and context).	Reported	p. 3, Introduction; p. 4, Methods 2.3	Objectives and Population-Concept-Context elements are explicit.
5	Indicate whether a review protocol exists; state if and where it can be accessed; and, if available, provide registration information, including the registration number.	Reported	p. 3, Methods 2.1; p. 15, Discussion 4.6	No protocol was prospectively registered; no retrospective registration was undertaken or is planned.
6	Specify characteristics of the sources of evidence used as eligibility criteria, and provide a rationale.	Reported	p. 4, Methods 2.3	Source characteristics, first-response boundary and English full-text restriction are specified.
7	Describe all information sources in the search and the date the most recent search was executed.	Reported	p. 4, Methods 2.2; Supplementary Section S1	Original and supplementary sources and search dates are reported.
8	Present the full electronic search strategy for at least one database, including any limits used, such that it could be repeated.	Reported	Supplementary Tables S1-S2	Full reproducible strings/query sets and limits are supplied.
9	State the process for selecting sources of evidence (i.e., screening and eligibility).	Reported	p. 4, Methods 2.4; pp. 5-6, Results 3.1 and Figure 1; Supplementary Section S7	Original and supplementary selection processes and exclusion reasons are described.
10	Describe the methods of charting data from the included sources of evidence (e.g., calibrated forms, whether charting was done independently or in duplicate) and any processes for obtaining and confirming data from investigators.	Reported	p. 5, Methods 2.5; Supplementary Section S3	Single-reviewer charting, second-reviewer checking and final consensus are stated.
11	List and define all variables for which data were sought and any assumptions and simplifications made.	Reported	p. 5, Methods 2.5; Supplementary Section S3	All charted variables and interpretive fields are listed.
12	If done, provide a rationale for conducting critical appraisal of included sources of evidence; describe the methods used and how this information was used in any data synthesis.	Not undertaken	p. 5, Methods 2.5	Formal risk-of-bias/quality appraisal was not undertaken; M0-M3 is not a critical-appraisal instrument.
13	Describe the methods of handling and summarising the data that were charted.	Reported	p. 5, Methods 2.5	Descriptive evidence mapping and maturity staging are explained.
14	Give numbers of sources of evidence screened, assessed for eligibility, and included in the review, with reasons for exclusions at each stage, ideally using a flow diagram.	Reported	pp. 5-6, Results 3.1 and Figure 1; Supplementary Section S8	Numbers screened, assessed and included are reported.
15	For each source of evidence, present characteristics for which data were charted and provide the citations.	Reported	pp. 7-9, Table 1; Supplementary Tables S4a-S4b	Source-level characteristics are presented with citations.
16	If done, present data on critical appraisal of included sources of evidence.	Not applicable	p. 5, Methods 2.5	No formal critical appraisal was conducted.
17	For each included source of evidence, present the relevant data that were charted and that relate to the review questions and objectives.	Reported	pp. 6-12, Results 3.2-3.8; Supplementary Tables S4a-S4b	Relevant source-level data and limitations are presented.
18	Summarise and/or present the charting results as they relate to the review questions and objectives.	Reported	pp. 5-12, Results; Tables 1-2	Charted findings are synthesised by application domain and evaluation depth.
19	Summarise the main results (including an overview of concepts, themes, and types of evidence available), link to the review questions and objectives, and consider the relevance to key groups.	Reported	pp. 12-16, Discussion 4.1-4.6	Main concepts, evidence types and relevance to services/survivors are summarised.
20	Discuss the limitations of the scoping review process.	Reported	pp. 15-16, Discussion 4.6	Review-process and evidence-base limitations are described.
21	Provide a general interpretation of the results with respect to the review questions and objectives, as well as potential implications and/or next steps.	Reported	p. 16, Conclusions	Interpretation and next steps are linked to the review objectives.
22	Describe sources of funding for the included sources of evidence, as well as sources of funding for the scoping review. Describe the role of the funders of the scoping review.	Reported	p. 17, Funding; Supplementary Table S4b	Review funding and funder role are reported; primary-source funding is charted where available in Table S4b.

Section S3. Data charting form

The form was used for descriptive evidence mapping. It was not a formal quality-appraisal instrument. "Not reported" indicates that the information was not identified in the source during data charting; "not evaluated" indicates that the characteristic or outcome was not assessed.

Field	Description
Source ID / full citation	Identifier aligned with the final reference numbering and complete bibliographic record.
Year and document type	Publication year and empirical/technical/qualitative/mixed-methods/audit/protocol/conceptual classification.
Country/region and service setting	Country, region, platform or service context; pathway point.
Population/data source and sample size	Survivors, users, professionals, records, posts, cases, speech data, systems or other material and reported sample/dataset size.
AI approach	Rule-based NLP, classical ML, deep learning, LLM, chatbot, RAG, speech model or other method.
Intended function	Information, triage, screening, reporting, prioritisation, legal guidance, referral/routing, documentation or modelling.
First-response category/directness	Direct survivor-facing; hotline/service-adjacent; clinician-facing; social-triage; legal/support routing; indirect/enabling.
Evaluation design and findings	Technical validation, usability/acceptability, audit, qualitative implementation, protocol or conceptual evidence and principal results.
Internal/external validation	Nature of model validation, including independent or cross-platform testing where reported.
Usability/acceptability	User-facing or stakeholder evaluation and participant characteristics.
Human oversight and handover/escalation	Role of clinicians, advocates or specialists; whether handover/escalation was operationalised or only proposed.
Service/pathway outcomes	Referral uptake, escalation completion, response time, workload, safety events, re-contact or other downstream endpoints.
Privacy/data governance and digital trace safety	Retention, access, logging, deletion, covertness, quick-exit/no-history, device monitoring and coercive-control considerations.
Equity/language/cultural considerations	Language coverage, subgroup analysis, accessibility and cross-context portability.
Product/commercial status and funding	Named-system status, author commercial affiliations, competing interests and primary-source funding where explicitly reported; not formally appraised.
M0-M3 and rationale	Deepest reported evaluation level and concise justification.
Principal pathway-level limitation	Main reason the source cannot establish real-world first-response effectiveness or safety.

Section S4. Detailed extraction matrices

Tables S4a-S4b report the complete charted fields in separate panels to preserve legibility. The panels should be read together. Product names are included only to identify the evaluated source and do not imply endorsement.

Table S4a. Source characteristics and first-response relevance — Part 1A: source and sample characteristics (sources 1-14).

Source	Year	Country/region	Service setting	Document type	Population/data source	Sample/dataset size
Vogt et al. [11]	2026	Austria, Germany, Finland, France and Spain; user survey in Germany	Survivor-facing domestic-violence information, safety-guidance and service-navigation chatbot	Mixed-methods needs-assessment and usability/acceptability study	Domestic-violence survivors and chatbot users	80 survivor interviews; 669-user survey
Socatiyanurak et al. [12]	2021	Thailand	Survivor-facing legal guidance after sexual violence	Model-development and technical-validation study	Thai Supreme Court sexual-violence decisions and legal-expert mock-up dialogues	182 Supreme Court cases; expert-generated dialogue set
Poveda et al. [13]	2026	Audit spanning chatbots available in 22 countries	Deployed domestic-violence chatbot ecosystem	Systems audit with user-interface/user-experience, privacy-policy and scenario-based evaluation	Publicly available domestic-violence chatbots	36 functional chatbots; 9 English/Spanish LLM web chatbots in scenario audit
Maeng and Lee [14]	2021	Republic of Korea	Design-stage sexual-violence support chatbot	Empirical exploratory question-analysis and technical-design study	Women asked to formulate questions from a survivor-positioned scenario	14 participants; 420 questions collected; 349 valid questions analysed
Maeng and Lee [15]	2022	Republic of Korea	First-contact information and emotional support after image-based sexual abuse	Prototype development and user study	Existing chatbot review, counsellor input and young adult women without reported image-based sexual abuse experience	9 chatbots reviewed; 5 counsellors; model test on 100 items; 25 user-study participants
Hossain et al. [16]	2020	Bangladesh	Domestic-violence mobile first-contact support during COVID-19	Prototype technical testing and usability evaluation	Domestic-violence case information and female usability participants	Domestic-violence data from Ain o Salish Kendra; 30 girls in System Usability Scale evaluation
Arrascue Casara et al. [17]	2023	Peru	Anonymous gender-violence/discrimination support and specialist appointment routing	Prototype validation with users and specialists	Women with violence experience and psychology specialists	25 women aged 15-50; 10 specialists
Wise [18]	2025	United States	National sexual-assault and domestic-violence crisis hotlines	Qualitative service-documentation study	Hotline communications and supporting documentation	Four national hotlines
Mielismäki and Husso [20]	2025	Finland	Domestic-violence services; ethics and governance for AI chatbots	Qualitative ethics and implementation study	Victim-survivors and professionals/stakeholders	25 interview participants
Tabaie et al. [29]	2022	United States	Emergency-department EHR identification of IPV encounters	Retrospective observational study with technical validation	Emergency-department encounters and clinical notes	1,064,735 ED encounters (2012-2020)
Sachdeva et al. [30]	2024	United States	ED prescreening for traumatic brain injury among IPV patients	Model-development and internal-validation study	Labelled clinical reports for IPV patients	71 positive and 122 negative cases
Majeed-Ariss et al. [31]	2025	United Kingdom	Sexual Assault Referral Centre triage at first contact	Prediction-model development with internal validation	Child/adolescent sexual-assault cases attending a SARC	492 cases; age >11 years
Fedele et al. [32]	2023	France	Forensic medical-centre pathway after recent sexual assault	Protocol	People aged >15 years reporting sexual assault within 30 days	Planned multicentre longitudinal cohort; final sample not reported in protocol results
Yazıcıoğlu and Yalçın Sarıbey [33]	2025	Türkiye / conceptual service context	Proposed domestic-violence hotline workflow	Conceptual perspective informed by secondary literature	No primary dataset; illustrative cases	Not applicable

Table S4a (continued) — Part 1B: source and sample characteristics (sources 15-27).

Source	Year	Country/region	Service setting	Document type	Population/data source	Sample/dataset size
Subramani et al. [34]	2018	Online/Facebook; service jurisdiction not specified	Domestic-violence social-media prioritisation for crisis services	Technical feasibility study	Public domestic-violence-related Facebook posts	2,060 labelled posts (750 critical; 1,310 uncritical)
Guan et al. [35]	2025	Online health communities; platform jurisdiction not specified	Professional triage of survivors' information needs	Prediction-model development and evaluation study	Online posts by adult women	294 real posts; 2,216 augmented instances; 74-post second-platform validation set
Sanz Urquijo et al. [36]	2025	Spain	Comparative evaluation of first-line GBV chatbot responses	Mixed-methods survivor-informed comparative evaluation	Survivors and scenario-generated chatbot responses	30 survivor interviews; scenario testing of general-purpose and domain-specific chatbots
Mutinda and Muchiri [37]	2024	Kenya	Bilingual GBV counselling chatbot	Technical feasibility study	Counselling content/training examples; no end-user sample reported	2,128 counselling question-response pairs used for model development and testing
Awasekar and Lobo [38]	2025	India (Maharashtra)	Domestic-violence awareness and legal-support chatbot	Prototype description with preliminary user feedback	Domestic-abuse victims	27 users
Subramani et al. [39]	2019	Online support groups; jurisdiction not specified	Categorisation of domestic-violence online posts for routing	Technical feasibility study	Domestic-violence-related online posts	1,654 labelled posts across five categories
Subramani et al. [40]	2017	Online/Facebook; jurisdiction not specified	Intent classification of domestic-violence discourse	Technical feasibility study	Facebook domestic-violence discourse	1,135 annotated posts (510 abuse; 625 advice/opinion), sampled from 8,856 posts and 28,873 comments
Reyner-Fuentes et al. [41]	2025	Spain / retrospective speech datasets	Speech-based GBV condition screening with privacy-oriented modelling	Model-development and validation study	Women represented in speech datasets	78 speakers at subject level
Barboza-Salerno et al. [42]	2026	United States	Emergency-department narrative surveillance of IPV injury visits	Retrospective longitudinal observational NLP surveillance study	NEISS emergency-department narratives	2013-2024 data; estimated 41,214 IPV-related ED visits
Gu et al. [43]	2026	United States	Clinical IPV risk identification before or during help-seeking	Model-development and validation study	Female patients from a domestic violence intervention/prevention centre and matched controls	Development cohort: 841 IPV cases and 5,212 controls; validation cohorts: 223 cases/1,333 controls, 95 cases/569 controls, and 186 cases/1,130 controls
Hui et al. [44]	2026	Hong Kong	Survivor-informed digital platform and AI chatbot design for help-seeking	Qualitative survivor-perspectives study	People with domestic-violence experience recruited via nongovernmental organisations/community channels	36 participants
Zhang et al. [45]	2026	Online forums; jurisdiction not specified	IPV disclosure analysis and potential social triage	Mixed qualitative and machine-learning analytic study	Women posting in IPV-related online forums	400 posts
Awasekar and Lobo [46]	2026	India	Survivor-facing domestic-violence legal/support-routing companion	Prototype/model evaluation with preliminary user/practitioner feedback	Legal evaluation set, one adjudicated Protection of Women from Domestic Violence Act case and research-preview users/practitioners	NyayaSmriti evaluation set; one case reference; preliminary feedback sample not fully reported

Table S4a (continued) — Part 2A: AI function and inclusion rationale (sources 1-14).

Source	AI approach	Intended function	First-response category	Directness	Inclusion rationale
Vogt et al. [11]	AinoAid™ domain-specific multilingual chatbot using structured dialogue/NLP	Tailored information, risk/safety guidance and navigation to support services	Direct survivor-facing first-line support	Direct	Evaluates a survivor-facing gateway explicitly positioned at entry to support pathways
Socatiyanurak et al. [12]	NLP/text-similarity chatbot with keyword and synonym matching	Match user-described incidents to relevant decisions and provide legal orientation	Legal/support routing	Direct survivor-facing prototype	Provides early legal guidance explicitly intended for sexual-violence victims/survivors
Poveda et al. [13]	Rule-based and LLM-powered chatbots	Audit privacy, covertness, boundaries and response quality in survivor-facing tools	Direct survivor-facing first-line support	Direct ecosystem audit	Audits deployed chatbots used by survivors at first contact
Maeng and Lee [14]	Proposed hybrid rule-based and machine-learning chatbot	Identify question patterns and inform conversational support design	Direct survivor-facing first-line support	Formative/design-stage	Uses empirical data to design a first-line conversational support system for sexual-violence survivors
Maeng and Lee [15]	Domain-specific chatbot with fine-tuned language model and structured support content	Provide immediate information, emotional support and support-seeking guidance	Direct survivor-facing first-line support	Direct prototype	Develops and evaluates a survivor-facing chatbot for an included form of sexual abuse
Hossain et al. [16]	Android app with NLP chatbot, emergency-alert action module and location functions	Provide information, rapid alerts, support contacts and initial assistance	Direct survivor-facing first-line support	Direct prototype	Combines an AI chatbot with first-contact reporting and emergency-alert functions for domestic violence
Arrascue Casara et al. [17]	Cloud platform using Amazon Lex conversational AI, web/Facebook interfaces	Anonymous communication, information provision and specialist scheduling	Direct survivor-facing first-line support	Direct prototype	Provides anonymous first-contact support and routing to specialists for gender violence
Wise [18]	LLM-powered chatbots as described by service providers	Characterise chatbot framing, scope boundaries and anthropomorphism	Hotline or service-adjacent support	Service-adjacent qualitative evidence	Examines how first-response services position chatbots to callers
Mielismäki and Husso [20]	AI-driven chatbots; technology-agnostic	Identify ethical, legal and operational deployment conditions	Hotline or service-adjacent support	Service-adjacent implementation evidence	Addresses governance and escalation requirements for first-response domestic violence chatbot deployment
Tabaie et al. [29]	Rule-based NLP with negation/history detection	Identify IPV encounters beyond administrative coding to prompt screening/referral	Clinician-facing first-contact decision support	Direct clinician-facing	Designed to support first-contact clinical identification and referral
Sachdeva et al. [30]	Supervised machine-learning ensemble using clinical-text features	Flag probable traumatic brain injury for further assessment and referral	Clinician-facing first-contact decision support	Direct clinician-facing	Supports acute first-contact assessment in an IPV clinical pathway
Majeed-Ariss et al. [31]	Logistic-regression prediction model	Identify pre-existing mental-health difficulties to support proactive referral	Clinician-facing first-contact decision support	Direct specialist-service triage	Targets referral planning at first contact in a sexual-assault service
Fedele et al. [32]	Planned predictive algorithms and trajectory modelling	Model psycho-social-judicial trajectories and PTSD risk to tailor follow-up	Clinician-facing first-contact decision support	Planned pathway integration	Protocol directly addresses early forensic/clinical pathway tailoring after sexual assault
Yazıcıoğlu and Yalçın Sarıbey [33]	Proposed NLP, speech sentiment analysis and predictive analytics	Screen, prioritise, escalate and document hotline contacts	Hotline or service-adjacent support	Conceptual	Directly proposes an AI-enabled first-response hotline workflow

Table S4a (continued) — Part 2B: AI function and inclusion rationale (sources 15-27).

Source	AI approach	Intended function	First-response category	Directness	Inclusion rationale
Subramani et al. [34]	Deep-learning binary text classification	Classify posts as critical/non-critical for rapid attention	Social-triage or online-disclosure support	Indirect/service-adjacent	Explicitly framed as prioritisation support for crisis services
Guan et al. [35]	Fine-tuned GPT-3.5 multi-class classifier	Classify eight information-need categories including safety planning, shelter and legal support	Social-triage or online-disclosure support	Service-adjacent decision support	Designed to help professionals tailor timely responses to survivor posts
Sanz Urquijo et al. [36]	ChatGPT, LLaMA and AinoAid comparative response evaluation	Assess empathy, contextual quality, bias and data responsibility	Direct survivor-facing first-line support	Direct response-quality evaluation	Evaluates AI responses intended for first-line GBV support using survivor-informed scenarios
Mutinda and Muchiri [37]	Rasa intent classification/dialogue management with fuzzy matching	Provide counselling-oriented information and referral in Kiswahili and English	Direct survivor-facing first-line support	Direct prototype	Explicitly intended as first-contact support when human services are unavailable
Awasekar and Lobo [38]	NLP/rule-based chatbot with legal/support content	Provide awareness, legal information and service navigation	Direct survivor-facing first-line support	Direct prototype	Survivor-facing information and navigation at early help-seeking
Subramani et al. [39]	Deep-learning multi-class text classification	Assign posts to needs/post categories for near-real-time routing	Social-triage or online-disclosure support	Indirect/service-adjacent	Explicitly links online-post classification to routing of needs
Subramani et al. [40]	Classical machine learning with feature engineering	Distinguish disclosure/help-seeking intent to support timely response	Social-triage or online-disclosure support	Indirect/service-adjacent	Framed as early triage to enable resources and response
Reyner-Fuentes et al. [41]	Domain-adversarial speaker-agnostic modelling	Classify a GBV-related victim condition while reducing speaker re-identification	Indirect or enabling first-response relevance	Enabling modality	Explicitly positioned as a potential screening aid for helplines/telehealth
Barboza-Salerno et al. [42]	Rule-based NLP plus survey-weighted estimates/regression	Identify IPV-related visits and contextual injury patterns beyond billing codes	Clinician-facing first-contact decision support	Service-system surveillance support	Supports ED screening, safety planning and prevention at first contact
Gu et al. [43]	Structured-data, clinical-note and multimodal Holistic AI in Medicine-style models	Identify patients at risk and support earlier connection to health/social resources	Clinician-facing first-contact decision support	Clinician-facing	Explicitly intended for earlier clinical identification and connection to support
Hui et al. [44]	Preferences for AI chatbot/digital platform features; no deployed model	Inform safe, accessible and culturally sensitive digital help-seeking design	Direct survivor-facing first-line support	Formative survivor-centred design	Directly examines survivor requirements for first-line AI-assisted help-seeking
Zhang et al. [45]	Qualitative coding, Random Forest/Neural Network classifiers and latent Dirichlet allocation topic modelling	Classify IPV subtypes and identify contextual patterns that may inform intervention design	Social-triage or online-disclosure support	Indirect/analytic	Explicitly links disclosure analysis to timely intervention development
Awasekar and Lobo [46]	Retrieval-augmented statute-aligned LAMP² 4.0 pipeline and SWATI interface	Provide legal literacy, relief prediction, indicative duration and next-step guidance	Legal/support routing	Direct survivor-facing prototype	Provides early legal/support orientation for domestic-violence survivors

Table S4b. Evaluation, implementation, safety and evidence interpretation — Part 1A: evaluation and findings (sources 1-14).

Source	Evaluation design	Main reported outcomes/findings	Internal validation	External validation	Usability/acceptability
Vogt et al. [11]	Qualitative needs assessment plus user survey	Generally positive openness, usability and perceived safety; users requested greater empathy, follow-up, personalisation and actionable links	Not applicable (user study)	No pathway-level external evaluation	Yes; interviews and large user survey
Socatiyanurak et al. [12]	Model development with cross-validation and hold-out testing	Combined approach reported approximately 88% top-1 accuracy on the hold-out set	Yes; cross-validation and hold-out testing	No independent external validation	No real-user usability evaluation
Poveda et al. [13]	Public-information review, privacy-policy analysis, usability audit and prompt scenarios	Substantial variation in privacy disclosures, covertness, boundaries and contextual response quality; several systems lacked adequate policies or safe responses	Not applicable (systems audit)	Multi-system, multi-country audit; not a pathway effectiveness evaluation	Interface/usability features audited; no survivor outcome study
Maeng and Lee [14]	Qualitative/quantitative question analysis and design implications	Questions clustered into 13 categories; many omitted context or used keywords only, motivating hybrid dialogue design	Not applicable (formative analysis)	No	No deployed prototype usability evaluation
Maeng and Lee [15]	Design process, technical test and comparative user study	Model reported 95% test accuracy; chatbot outperformed web search on organisation, accessibility, concision and perceived emotional support	Yes; 100-item technical test	No independent external validation	Yes; 25-participant user study
Hossain et al. [16]	Performance testing and System Usability Scale survey	Reported response time of 8.64 ms and mean System Usability Scale score of 66.71	Yes; prototype performance testing	No	Yes; 30-participant System Usability Scale survey
Arrascue Casara et al. [17]	Prototype validation and user/specialist acceptance survey	Women reported understandable language and goal completion; specialists reported high interface acceptance and perceived facilitation of interaction	Not applicable (prototype acceptance study)	No	Yes; women and specialist feedback
Wise [18]	Qualitative content analysis	Services described similar information/referral purposes but differed in human-like framing and claimed capabilities	Not applicable	Not applicable	Not evaluated
Mielismäki and Husso [20]	Semi-structured interviews and thematic analysis	Duty of care, accountability, privacy, trust and explicit escalation pathways identified as preconditions	Not applicable	Not applicable	Not a deployed-system usability study
Tabaie et al. [29]	Retrospective validation against manual review	Reported high precision and identified encounters not captured by administrative codes	Yes; sampled manual review	No independent external validation	Not evaluated
Sachdeva et al. [30]	Internal model development and testing	High sensitivity/accuracy reported within the study dataset	Yes	No	Not evaluated
Majeed-Ariss et al. [31]	Retrospective cohort model development/internal validation	Modest discrimination limited immediate practical utility	Yes	No	Not evaluated
Fedele et al. [32]	Protocol; no evaluative results	No results reported	Not evaluated	Not evaluated	Not evaluated
Yazıcıoğlu and Yalçın Sarıbey [33]	No primary evaluation	Conceptual workflow and forensic/technological considerations	Not evaluated	Not evaluated	Not evaluated

Table S4b (continued) — Part 1B: evaluation and findings (sources 15-27).

Source	Evaluation design	Main reported outcomes/findings	Internal validation	External validation	Usability/acceptability
Subramani et al. [34]	Technical evaluation on labelled corpus	High corpus-specific classification accuracy reported	Yes; corpus-level testing	No	Not evaluated
Guan et al. [35]	Internal testing plus second-platform external validation	Strong internal performance but decline on the second platform, indicating domain shift	Yes	Yes; 74 posts from a second platform	Not evaluated
Sanz Urquijo et al. [36]	Qualitative survivor material plus NLP-derived response analysis	Models differed in empathy and contextual alignment; all showed limits in intersectional sensitivity and proactive privacy attention	Scenario-based comparative analysis	No service-level external validation	Survivor perspectives informed evaluation; no live deployment study
Mutinda and Muchiri [37]	Technical intent-classification evaluation	Confusion-matrix/confidence performance reported; no end-user outcomes	Yes	No	Not evaluated
Awasekar and Lobo [38]	Prototype description and preliminary feedback	Preliminary favourable feedback; limited methodological detail on effectiveness	Not clearly reported	No	Preliminary user feedback
Subramani et al. [39]	Technical corpus evaluation	High within-corpus performance reported	Yes	No	Not evaluated
Subramani et al. [40]	Technical classification evaluation	Moderate corpus-specific performance reported	Yes	No	Not evaluated
Reyner-Fuentes et al. [41]	Leave-one-speaker-out technical evaluation	Moderate user-level classification with reduced speaker-identification performance	Yes; leave-one-speaker-out	No independent service validation	Not evaluated
Barboza-Salerno et al. [42]	Retrospective narrative case ascertainment and national estimates	Identified injury patterns and estimated national burden; no live workflow evaluation	Narrative case-identification process evaluated within dataset	No prospective external validation	Not evaluated
Gu et al. [43]	Model development and validation across additional network cohorts	Multimodal model reported an area under the receiver operating characteristic curve of 0.88	Yes	Additional same-network cohorts; not independent external service validation	Not evaluated
Hui et al. [44]	Reflexive thematic analysis	Participants valued evidence-based information, stepwise guidance, accessibility and non-judgemental support; raised concerns about empathy, accuracy and personalisation	Not applicable	Not applicable	Preferences/acceptability evidence; no prototype testing
Zhang et al. [45]	Qualitative content analysis plus supervised/unsupervised modelling	harmonic-mean precision-recall scores of approximately 0.62-0.85 for subtype classification; contextual themes identified	Yes; within-corpus evaluation	No	Not evaluated
Awasekar and Lobo [46]	Prototype/model testing and research-preview feedback	Reported 81% relief-prediction accuracy and favourable preliminary perceptions	Yes; prototype/legal evaluation	No independent external validation	Preliminary survivor/practitioner perceptions

Table S4b (continued) — Part 2A: oversight, pathway outcomes and safety (sources 1-14).

Source	Human oversight	Handover/escalation	Service/pathway outcomes	Privacy/data governance	Digital trace safety	Device monitoring/coercive control	Equity/language/culture
Vogt et al. [11]	Positioned as an adjunct to professional services	Human support recommended for high-risk situations; actual handover not evaluated	Referral completion, escalation success and safety incidents not evaluated	European Union-hosted architecture, personally identifiable information removal and data-protection controls described	Privacy/data-flow features described; quick-exit/no-history performance not evaluated	Perpetrator misuse considered in design; device-monitoring outcomes not evaluated	Five-country needs assessment; multilingual system; survey evaluation centred on Germany
Socatiyanurak et al. [12]	Legal-professional support remains necessary	No tested handover or referral workflow	No deployed service or pathway outcomes	Interface avoids requesting full name, profession or gender; retention/governance not evaluated	Digital trace, deletion and device-level safety not evaluated	Not evaluated	Thai-language and Thai-law specific; cross-jurisdictional portability not evaluated
Poveda et al. [13]	Human-service referral and scope boundaries varied across tools	Referral language audited; actual escalation/handover not evaluated	No referral completion, service uptake or adverse-event outcomes	Privacy policies, third-party tracking and data practices explicitly audited	Covertness and trace risks examined; deletion/no-history controls inconsistently available	Relevant to abuser discovery and covert use; real-world device-monitoring outcomes not tested	English/Spanish LLM subset; broader linguistic and cultural performance not evaluated
Maeng and Lee [14]	Human support anticipated but not operationalised	Not evaluated	Not evaluated	Research data secured/de-identified; deployment governance not evaluated	Tool-level trace safety not evaluated	Not evaluated	Small Korean female sample; cultural and demographic portability not evaluated
Maeng and Lee [15]	Professional counselling support recommended	Human counsellor connection recommended but not tested as a service workflow	No referral completion or pathway outcome evaluation	Research-data protection reported; deployment data governance not fully evaluated	Device-level trace, deletion and quick-exit functions not evaluated	Not evaluated	Young Korean female non-survivor sample; single scenario limits generalisability
Hossain et al. [16]	Women-support division/contact network described	Emergency-alert and support contacts included; completion and responder workflow not evaluated	No live service, referral or safety-outcome evaluation	Geolocation and emergency-alert data involved; retention, access controls and governance not evaluated	Device traces, notification safety, deletion and quick-exit not evaluated	Device-monitoring/abuser-access risk not evaluated	Bangladesh-specific prototype; language, disability and cross-context portability not evaluated
Arrascue Casara et al. [17]	Psychology specialists remain central to care	Appointment scheduling available; actual attendance/completion not evaluated	No verified referral uptake, response-time or pathway outcomes	System described as anonymous/secure; Facebook/Amazon Web Services governance and retention not evaluated	On-device traces, deletion and covertness not evaluated	Not evaluated	Peruvian/Spanish-language context; age range broad; portability not evaluated
Wise [18]	Referral to trained human advocates emphasised	Stated in service communications; completion not evaluated	No service outcome evaluation	Not the primary analytic focus	Not evaluated	Not evaluated	United States national services; language/cultural variation not evaluated
Mielismäki and Husso [20]	Human responsibility and professional support emphasised	Required conceptually; not tested	Not evaluated	Privacy and data governance discussed as core conditions	Digital safety concerns discussed; implementation outcomes not evaluated	Coercive-control context considered conceptually	Stakeholder perspectives in Finland; cross-context portability not evaluated
Tabaie et al. [29]	Clinicians/social workers remain responsible	Referral is intended but not operationally evaluated	No prospective workflow or referral outcome evaluation	EHR data governance followed source setting; not a review outcome	Not applicable to survivor-facing device traces	Not evaluated	Single trauma-centre dataset; subgroup portability not established
Sachdeva et al. [30]	Clinician decision support; not autonomous	Further assessment intended; not tested as workflow	No pathway outcomes	Clinical data handling reported by source; not evaluated as implementation outcome	Not applicable to survivor-facing device traces	Not evaluated	Small labelled dataset; subgroup performance and portability not established
Majeed-Ariss et al. [31]	Clinical decision-making and referral remain human-led	Referral intended; not tested as automated handover	No prospective service outcome evaluation	Clinical-case governance not evaluated as an outcome	Not applicable to survivor-facing device traces	Not evaluated	Single-centre paediatric/adolescent cohort; broader portability limited
Fedele et al. [32]	Clinical/medico-legal teams intended to remain responsible	Planned pathway integration; not evaluated	Not evaluated	Protocol governance reported by source; implementation outcomes unavailable	Not applicable/ not evaluated	Not evaluated	Eligibility and multicentre context described; subgroup outcomes not yet available
Yazıcıoğlu and Yalçın Sarıbey [33]	Human escalation presented as essential	Proposed, not tested	Not evaluated	Discussed conceptually; not evaluated	Not evaluated	Not evaluated	Not evaluated

Table S4b (continued) — Part 2B: oversight, pathway outcomes and safety (sources 15-27).

Source	Human oversight	Handover/escalation	Service/pathway outcomes	Privacy/data governance	Digital trace safety	Device monitoring/coercive control	Equity/language/culture
Subramani et al. [34]	Human review/response implied	Not evaluated	No live service or routing outcomes	Public-platform data use; survivor consent/governance not evaluated as pathway issue	Not evaluated	Not evaluated	Platform-specific language/context; subgroup portability not established
Guan et al. [35]	Explicitly framed as professional decision support	No direct automated response or tested referral workflow	No downstream service outcomes	Online-post data governance described by source; trace safety not an evaluated outcome	Not evaluated for user-facing deployment	Not evaluated	Adult-women data; cross-platform degradation documented; subgroup/language evidence limited
Sanz Urquijo et al. [36]	Escalation to trained advocates and clear boundaries emphasised	Recommended; not tested	No referral uptake or pathway outcomes	Data responsibility/privacy assessed in responses	Proactive trace-safety support was limited; no device-level deployment evaluation	Not evaluated in live use	Survivor-informed Spanish context; intersectional limitations explicitly identified
Mutinda and Muchiri [37]	Human services remain necessary	Referral information described; escalation not empirically evaluated	No live service outcomes	Not reported	Not evaluated	Not evaluated	Bilingual Kiswahili/English design; broader cultural/accessibility testing not reported
Awasekar and Lobo [38]	Primarily informational; human support not formalised	Not evaluated	No service/pathway outcomes	Not reported	Not evaluated	Not evaluated	Regional Indian context; portability and accessibility not evaluated
Subramani et al. [39]	Human response implied	Not evaluated	No live routing outcomes	Platform-data ethics/consent not evaluated as pathway outcome	Not evaluated	Not evaluated	Platform/language portability not established
Subramani et al. [40]	Human moderation/support implied	Not evaluated	No live workflow outcomes	Not evaluated	Not evaluated	Not evaluated	Platform-specific and no subgroup portability evidence
Reyner-Fuentes et al. [41]	Professional interpretation required	Not evaluated	No helpline/telehealth deployment outcomes	Privacy-oriented model objective directly assessed	Reduces voice re-identification risk; storage/retention/device traces not evaluated	Not evaluated	Female speaker dataset; language/cross-context portability not established
Barboza-Salerno et al. [42]	Clinical response remains human-led	Not evaluated	No prospective screening/referral outcomes	National surveillance data; implementation privacy not evaluated	Not applicable to user-facing device traces	Not evaluated	Sex-based injury differences analysed; broader subgroup performance not reported
Gu et al. [43]	Clinician decision support; no autonomous action	No deployed escalation/referral workflow evaluated	No downstream service outcomes	Clinical data governance reported by source; deployment privacy not evaluated as outcome	Not applicable to survivor-facing device traces	Not evaluated	Female clinical population; subgroup and cross-system portability limited/not fully reported
Hui et al. [44]	AI should complement human support	Participants emphasised access to human assistance; not tested	Not evaluated	Safety/privacy concerns reported by participants	Digital trace controls not evaluated in a deployed system	Relevant concerns raised; device-monitoring mitigation not tested	Hong Kong cultural context and community recruitment; local accessibility needs considered
Zhang et al. [45]	Professional interpretation would be required	Not evaluated	No service linkage or pathway outcomes	Use of forum narratives; pathway-level privacy/consent not evaluated	Not evaluated	Not evaluated	Platform-specific data; cross-platform, linguistic and demographic portability not established
Awasekar and Lobo [46]	Legal/professional support remains necessary	No tested legal-service referral/handover	No healthcare or service/pathway outcomes	Not reported in sufficient detail	Not evaluated	Not evaluated	Indian Protection of Women from Domestic Violence Act-specific; language, jurisdictional and cultural portability limited

Table S4b (continued) — Part 3A: product/funding, maturity and principal limitation (sources 1-14).

Source	Product/commercial status	Funding/declared affiliations	Maturity	Maturity rationale	Principal pathway-level limitation
Vogt et al. [11]	Named AinoAid™ system developed within the IMPROVE project; one author affiliated with We Encourage Oy Ltd.; no competing interests declared; no endorsement by this review	European Union Horizon Innovation Action IMPROVE, Grant 101074010; open-access funding via Projekt DEAL	M1	User-facing needs and usability/acceptability evidence without workflow-integrated evaluation	No verified handover, referral uptake, service endpoint or adverse-event monitoring
Socatiyanurak et al. [12]	Named academic research prototype; no commercial affiliation reported	Not reported	M0	Technical model validation without user-facing or service-integrated evaluation	Jurisdiction-specific technical evidence; no survivor, workflow or downstream outcome evaluation
Poveda et al. [13]	Audit of multiple named systems; no product endorsement and no single product effectiveness claim	Not reported	M1	Deployed-system audit and interface evidence without workflow or pathway outcomes	Audit findings do not establish survivor safety, service benefit or adverse-event rates
Maeng and Lee [14]	Academic design concept; no commercial product evaluated	National Research Foundation of Korea grant funded by the Korean National Police Agency and Ministry of Science and ICT (NRF-2018M3E2A1081492)	M0	Empirical formative design evidence without an evaluated user-facing system	Small scenario-based sample and no deployed chatbot, service workflow or outcome evaluation
Maeng and Lee [15]	Academic prototype; no commercial affiliation reported	Ministry of Education, Republic of Korea, and National Research Foundation of Korea (NRF-2021S1A5B8096358)	M1	User-facing prototype and usability evidence without service integration	Non-survivor sample, single scenario and no live handover or downstream outcomes
Hossain et al. [16]	Academic mobile-app prototype; no commercial affiliation reported	No funding reported in the source	M1	User-facing prototype with usability data but no service integration	Small usability sample and no live pathway, escalation or privacy-safety evaluation
Arrascue Casara et al. [17]	Academic prototype using third-party platforms; platform use is not evidence of author commercial affiliation	Not reported	M1	User/stakeholder acceptance evidence without pathway-level evaluation	Small prototype sample and no verified handover, service uptake or digital-safety outcomes
Wise [18]	Multiple service chatbots described; no product endorsement	Not reported	M1	Qualitative implementation/boundary evidence without workflow outcomes	Document analysis cannot establish actual user expectations, safety or handover performance
Mielismäki and Husso [20]	Technology-agnostic; no named product promoted	Not reported	M1	Qualitative implementation/ethics evidence without live workflow testing	Stakeholder evidence does not demonstrate tool performance or service outcomes
Tabaie et al. [29]	No commercial product identified	Not reported	M0	Retrospective technical validation only	Single-centre retrospective model with no prospective workflow or service endpoints
Sachdeva et al. [30]	No commercial product identified	Not reported	M0	Internal model validation without user/workflow evaluation	Small retrospective dataset and no external or pathway validation
Majeed-Ariss et al. [31]	No commercial product identified	Not reported	M0	Internal prediction-model validation only	Single-centre retrospective model with modest discrimination and no pathway testing
Fedele et al. [32]	No commercial product identified	Not reported	M0	Protocol without evaluative results	No completed data, model performance, workflow or safety outcomes
Yazıcıoğlu and Yalçın Sarıbey [33]	No product evaluation	Not reported	M0	Conceptual pathway proposal without primary data	No empirical evidence of feasibility, safety, service benefit or escalation performance

Table S4b (continued) — Part 3B: product/funding, maturity and principal limitation (sources 15-27).

Source	Product/commercial status	Funding/declared affiliations	Maturity	Maturity rationale	Principal pathway-level limitation
Subramani et al. [34]	No commercial product evaluated	Not reported	M0	Technical classification only	Platform-specific retrospective corpus and no live service linkage
Guan et al. [35]	Uses GPT-3.5 research pipeline; no product endorsement	Not reported	M0	Model development/external validation without workflow integration	External performance decline and no live professional/service evaluation
Sanz Urquijo et al. [36]	Multiple named models compared descriptively; no endorsement of AinoAid or general-purpose models	IMPROVE project context reported; funding/commercial involvement not formally appraised by this review	M1	Survivor-informed user-facing response evaluation without service workflow outcomes	Scenario-based evaluation cannot establish real-world safety, escalation or service benefit
Mutinda and Muchiri [37]	Academic prototype; no commercial affiliation reported	Not reported	M0	Technical feasibility only	No survivor usability, escalation, privacy or pathway evaluation
Awasekar and Lobo [38]	Named academic prototype; commercial status not reported	Not reported	M1	Preliminary user feedback without workflow evaluation	Small sample and limited reporting of methods, safety and downstream outcomes
Subramani et al. [39]	No product evaluation	Not reported	M0	Technical classification only	No live service integration, external validation or survivor-centred outcomes
Subramani et al. [40]	No product evaluation	Not reported	M0	Technical classification only	Retrospective platform corpus with no service linkage or safety evaluation
Reyner-Fuentes et al. [41]	No commercial product evaluated	Not reported	M0	Technical privacy/model validation only	Small retrospective dataset and no service deployment, handover or safety outcomes
Barboza-Salerno et al. [42]	No commercial product identified	Not reported	M0	Retrospective surveillance/model-derived evidence only	No live workflow, clinician behaviour, referral or survivor outcome evaluation
Gu et al. [43]	No commercial product identified	Not reported	M0	Model development/validation without workflow integration	No prospective service integration, escalation performance or independent external validation
Hui et al. [44]	No commercial product evaluated	Not reported	M1	Qualitative survivor-informed implementation/design evidence	No deployed AI tool, workflow behaviour or service outcome evaluation
Zhang et al. [45]	No product evaluation	Not reported	M0	Analytic/model evidence without workflow integration	Small platform-specific corpus and no verified intervention or routing process
Awasekar and Lobo [46]	Named academic prototype; commercial status not reported; no endorsement by this review	Not reported	M1	Prototype performance plus preliminary user/practitioner feedback	Preliminary, jurisdiction-specific evidence without deployed service or safety outcomes

Section S5. Conceptual crosswalk for the M0-M3 descriptive heuristic

This crosswalk is explanatory only. It does not establish formal equivalence, validation or quality scoring, and the included sources were not fully recoded against IDEAL, DECIDE-AI or the NICE Evidence Standards Framework.

Table S5. Conceptual crosswalk between the author-developed M0-M3 heuristic and established staged-evaluation frameworks.

Level	Operational definition in this review	Broad relation to IDEAL	Broad relation to DECIDE-AI	Broad relation to NICE ESF	Limitations of comparison
M0	Technical development/validation only, protocol without results, or conceptual pathway proposal	Broad analogy to Idea/Development stages; not a direct application of the surgical framework	Usually pre-live evaluation and often outside DECIDE-AI's target of early clinical evaluation	Technical/analytical evidence supporting limited claims; insufficient for service-effectiveness claims	Framework scope differs across clinical, chatbot, legal and social-triage records; no formal equivalence.
M1	User-facing usability/acceptability, systems audit, preliminary feedback, or qualitative implementation/ethics evidence	Broad analogy to Development/Exploration and formative human-factors work	Partial overlap with human-factors and formative reporting; DECIDE-AI applies directly only to relevant live clinical decision-support evaluations	Usability, acceptability and early real-world evidence may support bounded claims, not pathway effectiveness	M1 combines heterogeneous formative evidence; it is not a quality rank.
M2	Workflow-integrated pilot with documented handover/escalation processes	Broad analogy to Exploration/early Assessment	Closest conceptual overlap with early live clinical/service evaluation and human-AI workflow reporting	Prospective workflow and real-world performance evidence becomes relevant to service claims	No included source reached M2; relation varies by service context.
M3	Pathway-level evaluation with service endpoints and safety/adverse-event monitoring	Broad analogy to Assessment and longer-term monitoring	Extends beyond early-stage DECIDE-AI towards comparative effectiveness and post-deployment monitoring	Higher-level real-world evidence, comparative outcomes and ongoing performance/safety monitoring	No included source reached M3; thresholds remain author-defined and require external refinement.

No independent inter-rater reliability test was conducted. Source-level assignments were performed using predefined decision rules, checked by a second reviewer, and resolved by final consensus. The crosswalk is intended to make the staging rationale transparent; it does not confer validation or evaluative authority.

Section S6. Source-level M0-M3 coding matrix

Table S6. Source-level M0-M3 coding matrix.

Source	Document/evidence type	Application domain	Maturity	Rationale
Vogt et al. [11]	Mixed-methods needs-assessment and usability/acceptability study	Direct survivor-facing first-line support	M1	User-facing needs and usability/acceptability evidence without workflow-integrated evaluation
Socatiyanurak et al. [12]	Model-development and technical-validation study	Legal/support routing	M0	Technical model validation without user-facing or service-integrated evaluation
Poveda et al. [13]	Systems audit with user-interface/user-experience, privacy-policy and scenario-based evaluation	Direct survivor-facing first-line support	M1	Deployed-system audit and interface evidence without workflow or pathway outcomes
Maeng and Lee [14]	Empirical exploratory question-analysis and technical-design study	Direct survivor-facing first-line support	M0	Empirical formative design evidence without an evaluated user-facing system
Maeng and Lee [15]	Prototype development and user study	Direct survivor-facing first-line support	M1	User-facing prototype and usability evidence without service integration
Hossain et al. [16]	Prototype technical testing and usability evaluation	Direct survivor-facing first-line support	M1	User-facing prototype with usability data but no service integration
Arrascue Casara et al. [17]	Prototype validation with users and specialists	Direct survivor-facing first-line support	M1	User/stakeholder acceptance evidence without pathway-level evaluation
Wise [18]	Qualitative service-documentation study	Hotline or service-adjacent support	M1	Qualitative implementation/boundary evidence without workflow outcomes
Mielismäki and Husso [20]	Qualitative ethics and implementation study	Hotline or service-adjacent support	M1	Qualitative implementation/ethics evidence without live workflow testing
Tabaie et al. [29]	Retrospective observational study with technical validation	Clinician-facing first-contact decision support	M0	Retrospective technical validation only
Sachdeva et al. [30]	Model-development and internal-validation study	Clinician-facing first-contact decision support	M0	Internal model validation without user/workflow evaluation
Majeed-Ariss et al. [31]	Prediction-model development with internal validation	Clinician-facing first-contact decision support	M0	Internal prediction-model validation only
Fedele et al. [32]	Protocol	Clinician-facing first-contact decision support	M0	Protocol without evaluative results
Yazıcıoğlu and Yalçın Sarıbey [33]	Conceptual perspective informed by secondary literature	Hotline or service-adjacent support	M0	Conceptual pathway proposal without primary data
Subramani et al. [34]	Technical feasibility study	Social-triage or online-disclosure support	M0	Technical classification only
Guan et al. [35]	Prediction-model development and evaluation study	Social-triage or online-disclosure support	M0	Model development/external validation without workflow integration
Sanz Urquijo et al. [36]	Mixed-methods survivor-informed comparative evaluation	Direct survivor-facing first-line support	M1	Survivor-informed user-facing response evaluation without service workflow outcomes
Mutinda and Muchiri [37]	Technical feasibility study	Direct survivor-facing first-line support	M0	Technical feasibility only
Awasekar and Lobo [38]	Prototype description with preliminary user feedback	Direct survivor-facing first-line support	M1	Preliminary user feedback without workflow evaluation
Subramani et al. [39]	Technical feasibility study	Social-triage or online-disclosure support	M0	Technical classification only
Subramani et al. [40]	Technical feasibility study	Social-triage or online-disclosure support	M0	Technical classification only
Reyner-Fuentes et al. [41]	Model-development and validation study	Indirect or enabling first-response relevance	M0	Technical privacy/model validation only
Barboza-Salerno et al. [42]	Retrospective longitudinal observational NLP surveillance study	Clinician-facing first-contact decision support	M0	Retrospective surveillance/model-derived evidence only
Gu et al. [43]	Model-development and validation study	Clinician-facing first-contact decision support	M0	Model development/validation without workflow integration
Hui et al. [44]	Qualitative survivor-perspectives study	Direct survivor-facing first-line support	M1	Qualitative survivor-informed implementation/design evidence
Zhang et al. [45]	Mixed qualitative and machine-learning analytic study	Social-triage or online-disclosure support	M0	Analytic/model evidence without workflow integration
Awasekar and Lobo [46]	Prototype/model evaluation with preliminary user/practitioner feedback	Legal/support routing	M1	Prototype performance plus preliminary user/practitioner feedback

Section S7. Supplementary full-text screening decisions

The 27 supplementary full texts were assessed using the same first-response eligibility boundary as the original review. “Background only” records informed interpretation but were not counted as included evidence.

Table S7. Supplementary full-text screening decisions.

Title or bibliographic identifier	Decision	Reason
Can a Conversational Agent Lower Sexual Violence Victims' Burden of Self-Disclosure?	Background only	Relevant design evidence, but no implemented or evaluated AI first-response tool; used to inform discussion of disclosure burden.
A Chatbot Solution to Chat App Problems for Teenage Victims of Online Sexual Exploitation	Background only	Design-guideline study from peer-counsellor interviews; no implemented/evaluated AI system; retained as adjacent design evidence.
Designing a Chatbot for Survivors of Sexual Violence	Included	Hybrid rule-based/machine-learning chatbot concept and analysis of survivor-positioned questions; directly relevant to first-line support.
Personalization Trade-offs in Designing a Dialogue-based Information System	Background only	Strong support-seeking design evidence, but the prototype is not clearly AI-enabled under the review concept.
Designing and Evaluating a Chatbot for Survivors of Image-Based Sexual Abuse	Included	Developed and evaluated a survivor-facing chatbot for immediate information and emotional support after image-based sexual abuse.
Trauma-Informed Design with a Data Donation Platform for Online Dating Sexual Violence	Background only	Concerns trauma-informed design and data donation for future AI, not a first-response support tool.
AI-Facilitated Coercive Control	Background only	Addresses AI as a potential perpetration/risk vector rather than AI-enabled survivor support.
Deepfake, Real Harm	Background only	Important for AI-enabled sexual abuse and response infrastructures, but not an evaluated first-response support tool.
(Re)mediators of Epistemic Injustice	Background only	Empirical work on generative AI and intimate partner violence sensemaking; not a defined first-response tool or service pathway.
Trauma-Informed Data Donation	Background only	Methodological/design infrastructure for sexual-violence data collection, not survivor first response.
Insights from Auditing AI Chatbots for Survivors of Domestic Violence	Included	Audit of deployed domestic violence chatbots, privacy, interface, and scenario responses; directly relevant to survivor-facing first-line tools.
In the Shadows of Transparency	Background only	AI-mediated crisis work focused on Crisis Text Line and concealment; not sufficiently specific to gender-based violence for inclusion.
Machine Learning and Community-Driven Feminist Cyber Resistance Framework	Excluded	Online content moderation/flagging framework; no survivor first-contact support, service routing, or pathway evaluation.
SecondLook dating abuse detection and awareness app	Excluded	Machine-learning-enabled dating-abuse awareness/detection app; prevention/awareness rather than post-violence first-response support.
DistilBERT-based anonymous complaint reporting	Excluded	Broad abuse-reporting simulation across child abuse, domestic violence and harassment; not specifically linked to a gender-based-violence or sexual-violence response pathway.
Artificial Intelligence Based Legal Application for Combating Domestic Violence	Excluded	Conceptual proposal with limited implementation/evaluation detail; more mature legal-support records retained.
Combating Domestic Violence during COVID-19 Pandemic in Bangladesh	Included	Natural language processing chatbot/emergency-alert mobile app with performance and System Usability Scale evaluation; first-contact domestic violence support.
Designing Human-centered AI to Assist with Domestic Abuse Recovery	Background only	Addresses recovery and technology-enabled coercive control rather than early first-response support.
Knowledge-aware response generation in clinical counselling/legal support	Excluded	General counselling/legal-support model; not specific to sexual-violence or gender-based-violence first-response pathways.
Gender Violence on Arabic Facebook	Excluded	Text mining of discourse; no support, routing or first-response intervention.
Hierarchical RAG for Multimodalized Woman's Companion	Excluded	Women's-health chatbot with a broad sexual and reproductive health focus; not specific to gender-based-violence or sexual-violence first-response pathways.
Legal Guidance Through AI Chatbot for Sexual Violence Victims and Survivors	Included	Survivor-facing legal guidance chatbot for sexual-violence cases with natural language processing model validation.
Meta-analysis of AI Solution for Prevention of Violence Against Women and Girls	Excluded	Secondary synthesis; used only to check context, not eligible as primary evidence.
NLP through BERT for Identifying GBV Messages on Social Media	Excluded	Model-level social-media classification without service linkage or support/routing function.
Advancing AI Models to Assist Victims of Domestic Abuse	Duplicate/overlap	Overlaps substantially with the more complete NyayaSakhi-SWATI record already included; not counted separately.
Specialist-Woman Interaction System	Included	Cloud platform and conversational assistant for anonymous communication and specialist scheduling.
Transformers for Supreme Court Decisions Related to Sexual Violence Law	Background only	Technical legal natural language processing component related to LAW-U; no direct survivor-facing pathway evaluation.

Section S8. Final search record accounting

Original and supplementary streams are reported separately because the targeted search was conducted after the original database searches. IEEE counts reflect within-source deduplication. ACM records/candidates were screened in-platform; potentially eligible records were retained for full-text assessment, and residual overlap between sources and with the original corpus was resolved during screening.

Table S8. Final search record accounting.

Item	Count/decision
Original records identified through database searching	187
Original duplicates removed	90
Original records screened after deduplication	97
Original records excluded at title/abstract	74
Original reports sought for retrieval	23
Original reports not retrieved	1
Original full texts assessed	22
Original full texts excluded	1 systematic review, used only for contextual discussion
Original sources included	21
IEEE Xplore supplementary records after within-source deduplication	123
ACM Digital Library supplementary records/candidates screened in-platform	416
Total supplementary records/candidates screened	539; residual overlaps between sources and with the original corpus were resolved during screening
Supplementary records excluded at title/abstract/in-platform screening	512
Supplementary full texts retrieved and assessed	27
Supplementary full texts not included in the final synthesis	21
— Background only	11
— Excluded as ineligible	9
— Duplicate/overlap	1
Supplementary full texts included	6
Final sources included in scoping synthesis	27
